# Education debt and household consumption upgrading: Positive incentives or inhibitions?

**DOI:** 10.1371/journal.pone.0332318

**Published:** 2025-10-13

**Authors:** Mianbi Xie, Xin Chen, Yingying Zhao

**Affiliations:** College of Finance and Economics, Jimei University, Xiamen, China; U of U: University of Utah, UNITED STATES OF AMERICA

## Abstract

Household education debt is closely related to household consumption, and education itself is also a developmental high-quality consumption. Based on the panel data of five phases of the China Household Finance Survey (CHFS) from 2013 to 2021, this paper explores the impact of households’ education debt on improving household consumption enthusiasm and consumption upgrading with households as the basic unit. The study finds that education debt can significantly promote the upgrading of household consumption. Mechanism analysis shows that education debt can promote the upgrading of household consumption by improving the level of Internet consumption, and there are different degrees of moderating effect on household risk attitude. Heterogeneity analysis shows that household education debt has a significant positive effect on the consumption of urban and rural areas, high-financially literate and middle- and low-income households, as well as middle-leveraged households. The conclusions of this study enrich the research on the influencing factors and mechanisms of household consumption upgrading, broaden the research boundary of household debt and consumption, and have important implications for promoting education equity and consumer demand in China.

## 1. Introduction

In the context of economic globalization and the knowledge-based economy, education is widely recognized as a key driver of socio-economic development and a major factor in enhancing individual competitiveness. China’s recent “double-reduction” policy, which aims to reduce the burden of extracurricular and out-of-school training for students, has significantly reshaped household investment and consumption patterns in education [1]. By curbing out-of-school training, the policy has reduced households’ additional education expenses, freeing up disposable income for other consumption areas and promoting consumption upgrading. However, as national policies continue to evolve, the education and training industry is gradually recovering. The State Council’s guidelines now advocate leveraging high-quality educational resources to meet diverse learning needs and encourage social training institutions to enhance service quality.

Despite these changes, rising education costs have led to an increase in household education debt. As a critical investment in future development, education debt has significant implications for household consumption structures and consumption upgrading [2]. According to data from the National Bureau of Statistics of China, the total retail sales of consumer goods in China have grown dramatically from 155.86 billion yuan in 1978–47 trillion yuan in 2023, with an average annual increase of 7.2%. This growth reflects China’s position as the world’s second-largest consumer market. However, the consumption rate of Chinese residents remains low, with insufficient domestic demand constraining further economic growth. Since the 18th National Congress of the Communist Party of China, education has become the largest category of public budget expenditure, accounting for over 4% of GDP. Against this backdrop, the “double-reduction” policy aims to optimize education-related expenditures, allowing household to reallocate savings toward improving quality of life in areas such as health, tourism, and cultural entertainment.

In recent years, education has gained increasing attention for its role in enhancing human capital and driving high-quality economic growth [3]. China’s Strategic Plan for Domestic Demand Expansion (2022–2035) prioritizes expanding domestic demand and consumption. Educational investment through debt has become a key aspect of household financial decisions, influencing both household economic well-being and consumption behavior. The 20th National Congress of the Communist Party of China further integrated education, science, and human resources into a cohesive strategy. As a long-term investment, education debt can improve household consumption by enhancing education levels and future income potential [4]. However, the relationship between education and high-quality consumption, especially regarding education debt and household consumption upgrading, remains underexplored. This study examines how household education debt stimulates consumption enthusiasm and potential. By analyzing the role of household consumption in China’s economic development, we construct a theoretical framework and conduct empirical research to elucidate the interplay between education debt and consumption upgrading. Our findings offer insights into optimizing consumption structure and promoting sustainable economic growth.

The main contributions and innovations of this study are as follows, and the flow chart of this research is shown in following S1 Fig.

This study makes significant theoretical breakthroughs by innovatively incorporating household education expenditure and education debt variables into the classic Life Cycle-Permanent Income Hypothesis (LC-PIH) framework, thereby expanding the theoretical boundaries of traditional intertemporal consumption models. By integrating the precautionary savings theory, we systematically reveal the crucial moderating role of household risk expectations in the relationship between education debt and consumption behavior. This theoretical innovation not only addresses the research gap in existing literature that overemphasizes housing and medical debt while neglecting education debt, but also provides a novel theoretical perspective and analytical framework for understanding household consumption decision-making mechanisms.

In terms of empirical research design, this study utilizes five-wave tracking data (2013–2021) from the China Household Finance Survey (CHFS), covering a nationally representative sample of households across 29 provinces (autonomous regions/municipalities). This high-quality panel data effectively controls for individual heterogeneity while capturing temporal trends. Through the application of a two-way fixed effects model, we significantly reduce omitted variable bias, thereby providing robust data support for accurately identifying the causal relationship between education debt and household consumption upgrading.

At the mechanism analysis level, this study makes three prominent contributions: First, it innovatively introduces internet consumption as a mediating variable, revealing new pathways through which education debt affects household consumption in the digital economy era. Second, it systematically examines the moderating effect of household risk attitudes for the first time, deepening our understanding of behavioral heterogeneity. Finally, through multidimensional subgroup analysis (income levels, urban-rural differences, financial literacy, and debt levels), it provides precise policy targets for formulating differentiated education credit policies and consumption stimulation measures. These findings not only expand the research dimensions of household consumption theory, but also provide important empirical evidence and policy insights for promoting the virtuous interaction between education investment and consumption upgrading.

## 2. Literature review

This paper draws on two key strands of literature. The first strand of literature examines how education debt affects household consumption structure. Scholars have clarified that education debt refer to debts incurred by household to finance members’ education. These debts are categorized into two types: formal loans from financial institutions (e.g., national and commercial student loans) and informal borrowings from private sources (e.g., relatives, friends, or private lenders) [5–8]. In China, rising household education expenditures, particularly for extracurricular activities and overseas studies [9,10], have increased education debt. This not only reduces overall household consumption capacity but also subtly alters consumption structure [11]. Moreover, education debt may impair households’ ability to establish emergency funds, which are crucial for economic resilience during shocks [12]. Since the implementation of China’s parallel fee system for higher education, educational expenditures and debt have surged. The literature reviews the education loan crisis, analyzing trends in tuition fees, student debt growth, and their profound impacts on individuals and society [13].

The second strand of literature examines factors influencing household consumption. Household consumption levels and structure are shaped by both internal and external factors. Internally, individual characteristics and overall household conditions—such as education, age distribution income, assets, and debt levels—play a crucial role [14–18]. And Barro and Lee (2013) updated and expanded the panel dataset on educational attainment, providing robust data support for investigating the impact of education levels on economic and social variables [19]. According to the persistent income hypothesis, income growth and asset accumulation are key drivers of higher consumption levels. Household asset accumulation directly impacts consumption through the wealth effect [20,21]. Additionally, changes in household age structure, particularly increases in the elderly dependency ratio [22], optimize consumption structure by shifting spending from basic necessities to healthcare and recreation, but the impact of the child dependency ratio is also complex and ambiguous [23].

Externally, advancements in digital technology, financial market development, and macroeconomic fluctuations also significantly influence consumption trends. For instance, the widespread adoption of digital technologies like the Internet and artificial intelligence drives consumption upgrading and creates new trends [24]. Financial market development provides consumers with liquidity constraints a range of financial tools, facilitating effective intertemporal consumption planning and stimulating latent demand [25]. However, macroeconomic volatility, such as China’s economic slowdown in 2013, directly impacts consumption behavior, especially in service consumption and housing expenditure [26]. And Liu et al (2019). point out that as household homeownership rates increase, the wealth effect gradually strengthens while the crowding-out effect weakens. Consumption upgrading involves increased total consumption, improved quality, and new forms of consumption [27–28]. Overall, household consumption is a product of multiple interacting factors that collectively shape consumption decisions and behaviors through complex mechanisms.

In summary, limited research has explored education debt as a driver of household consumption upgrading. Theoretically, the impact of education debt on household consumption expenditures remains unclear, and analyses of their role in consumption structure and upgrading are insufficient. This study addresses this gap by examining the potential effects of education debt on household consumption upgrading through a theoretical lens and constructing a benchmark regression model at the household level. This approach aims to enrich the theoretical understanding of the relationship between education debt and household consumption and provide insights for promoting rational consumption upgrading and balanced economic development.

## 3. Theoretical modeling and research inferences

Education is crucial for stimulating consumer vitality and expanding domestic demand [29]. Building on the life cycle-permanent income hypothesis and the precautionary savings theory, this paper integrates household education expenditure $E_{t}$ and liabilities $D_{E}$ into an intertemporal consumption model [30–33]. This framework explores how education debt affects household consumption and the underlying mechanisms. Assuming that each household begins at period 0 and has an infinite time horizon, the household’s intertemporal utility maximization function can be expressed as:


$$\begin{array}{c} U_{max}=\sum_{t=\text{0}}^{\infty }\beta^{t}U\left(C_{t}\right) \end{array}$$ (1)

Where U(·) denotes the household utility function, satisfy $U^{\prime }(\cdot )>\text{0}$ and $U^{\prime \prime }(\cdot )<\text{0}$; $C_{t}$ denotes total household consumption expenditure; β denotes the utility discount factor, which satisfies 0<β<1.

Hou and Fu (2023) pointed out that there are a short-term consumption effect and a long-term investment effect in household education expenditure [34]. According to the short-term consumption effect, the household’s education expenditure in period t belongs to the developmental consumption expenditure. In order to simplify the model, this paper regards education expenditure as developmental consumption expenditure, then the total household consumption expenditure can be expressed as:


$$C_{t}=C_{s,t}+C_{e,t}+E_{t}$$

Where $C_{s,t}$ and $C_{e,t}$ denote subsistence and enjoyment consumption expenditures, respectively. Assuming that there is always no education expenditure, the household’s persistent income in the t period is $Y_{t}$. According to the long-term investment effect of education, if the household invests $E_{t-\text{1}}$ in education in the (t−1) period, the persistent income in the t period will be raised $f\left(E_{t-\text{1}}\right)$ accordingly, and the raised income will be $Y_{t}\cdot f\left(E_{t-\text{1}}\right)$. That is to say, education itself has a certain cumulative effect, and its effect on the improvement of income has a long-lasting nature, which is manifested in the following: $Y_{t+\text{1}}=Y_{t}\cdot f\left(E_{t-\text{1}}\right)$.

### 3.1 Disregarding investment decisions

It is constrained without considering the investment behavior of the household:


$$\begin{array}{c} \begin{array}{c} W_{t+\text{1}}=(\text{1}+r)\left[W_{t}+Y_{t}\cdot f\left(E_{t-\text{1}}\right)-C_{t}\right]\\ \;\;\;\;\;\;\;\;=(\text{1}+r)\left[W_{t}+Y_{t}\cdot f\left(E_{t-\text{1}}\right)-C_{s,t}-C_{e,t}-E_{t}\right] \end{array} \end{array}$$ (2)

where $W_{t}$ denotes the household’s level of wealth at the beginning of period *t*; r denotes the return on assets and is assumed to remain constant across periods.

According to equations (1) and (2), the Lagrangian function is established as:


$$\begin{array}{c} \begin{array}{c} L=\sum_{t=\text{0}}^{\infty }\left\{\beta^{t}U\left(C_{t}\right)+\lambda_{t}\left[(\text{1}+r)\;\left(W_{t}+Y_{t}\cdot f\left(E_{t-\text{1}}\right)-C_{t}\right)\;-W_{t+\text{1}}\right]\right\}\\ \;\;\;\;\;\;\;=\sum_{t=\text{0}}^{\infty }\left\{\beta^{t}U\left(C_{t}\right)+\lambda_{t}\left[(\text{1}+r)\;\left(W_{t}+Y_{t}\cdot f\left(E_{t-\text{1}}\right)-C_{s,t}-C_{e,t}-E_{t}\right)\;-W_{t+\text{1}}\right]\right\} \end{array} \end{array}$$ (3)

where $\lambda_{t}$ is the Lagrange multiplier. Next, the system of first order conditional equations to be satisfied in order to solve the optimization problem is:


$$\left\{ \begin{array}{c} \frac{\partial L}{\partial C_{t}}=\beta^{t}U^{\prime }\left(C_{t}\right)-(\text{1}+r)\lambda_{t}=\text{0}\\ \frac{\partial L}{\partial W_{t}}=-\lambda_{t-\text{1}}+(\text{1}+r)\lambda_{t}=\text{0}\\ \frac{\partial L}{\partial E_{t}}=-(\text{1}+r)\lambda_{t}+(\text{1}+r)\lambda_{t+\text{1}}Y_{t+\text{1}}\frac{df\left(E_{t}\right)}{dE_{t}}=\text{0} \end{array}\right.\;$$ (4)

This can be obtained by solving using Euler’s equation:


$$\begin{array}{c} U^{\prime }\left(C_{t}\right)=\beta (\text{1}+r)U^{\prime }\left(C_{t+\text{1}}\right) \end{array}$$ (5)

$$\begin{array}{c} U^{\prime }\left(C_{t}\right)=\beta Y_{t+\text{1}}\frac{df\left(E_{t}\right)}{dE_{t}}U^{\prime }\left(C_{t+\text{1}}\right) \end{array}$$ (6)

To simplify the model, it is assumed that education expenditures are equal $\left(E_{t}=E_{t-\text{1}}=E\right)$ in all periods. According to equations (5) and (6):


$$\begin{array}{c} \frac{df(E)}{dE}f(E)=\frac{\text{1}+r}{Y_{t}} \end{array}$$ (7)

Further integrating and simplifying equation (7) yields the following revenue enhancement function:


$$\begin{array}{c} f(E)=\sqrt{\frac{\text{2}(\text{1}+r)E}{Y_{t}}}+\varepsilon_{\text{1}} \end{array}$$ (8)

Where $\varepsilon_{\text{1}}$ is a constant term.

Substituting equation (8) into constraint (2), the solution yields the total consumption expenditure $C_{t}$ of the household in tperiod:


$$\begin{array}{c} C_{t}=\sqrt{\text{2}(\text{1}+r)Y_{t}E}+\varepsilon Y_{t}+W_{t}-\frac{W_{t+\text{1}}}{\text{1}+r} \end{array}$$ (9)

Further partial derivatives for education expenditures E:


$$\begin{array}{c} \frac{{\partial C}_{t}}{\partial E}=\sqrt{\frac{(\text{1}+r)Y_{t}}{\text{2}E}}>\text{0} \end{array}$$ (10)

Accordingly, it can be seen that household expenditure on education has a positive impact on their overall consumption expenditure. As China’s economy has boomed and its per capita GDP has jumped significantly, there has been a profound change in consumption attitudes, especially the growing demand for education services [35]. This trend not only pushes the growth of household investment in education, but also contributes to the upward trend of household investment in education debt. As an effective strategy for smoothing education expenditures [36], household education debt has boosted household education expenditures while also having an impact on their total consumption expenditures.

Therefore, this paper assumes that the proportion of the household’s education debt in period t as α percentage of the total educational expenditure in that period is fixed, i.e., the equation $D_{E}=\alpha E$ is satisfied, where 0<α≤1. Substituting this equation condition into the consumption function (9), we obtain the expression of the total consumption function including the household’s education debt:


$$\begin{array}{c} C_{t}=\sqrt{\frac{\text{2}(\text{1}+r)Y_{t}D_{E}}{\alpha }}+\varepsilon_{\text{1}}Y_{t}+W_{t}-\frac{W_{t+\text{1}}}{\text{1}+r} \end{array}$$

Take a partial derivative for the education debt $D_{E}$:


$$\begin{array}{c} \frac{{\partial C}_{t}}{\partial D_{E}}=\sqrt{\frac{(\text{1}+r)Y_{t}}{\text{2}\alpha D_{E}}}>\text{0} \end{array}$$ (11)

Combining equations (10) and (11), this paper compares the difference in consumption changes between the two scenarios of households choosing not to take on versus taking on education debt, and obtains the following inequality:


$$\text{0}<\frac{{\partial C}_{t}/\partial E}{{\partial C}_{t}/\partial D_{E}}=\frac{\sqrt{\frac{(\text{1}+r)Y_{t}}{\text{2}E}}}{\sqrt{\frac{(\text{1}+r)Y_{t}}{\text{2}\alpha D_{E}}}}=\sqrt{\frac{\alpha D_{E}}{E}}=\alpha \leq \text{1}$$

According to this inequality, for households with education debt, the impact of education expenditure on household consumption includes not only the impact of total education expenditure E on household consumption ($\frac{{\partial C}_{t}}{\partial E}$), but also the impact of education debt $D_{E}$ on household consumption ($\frac{{\partial C}_{t}}{\partial D_{E}}$). In other words, the increase in total consumption expenditures is more significant for households with education debt than for those without. This suggests that education debt effectively raises overall household consumption levels. Moreover, development and enjoyment consumption tend to have higher expenditure elasticity than subsistence consumption. When households take on education debt, it often reflects higher demands for education and a better cultural environment, leading to increased spending in these areas [37]. Therefore, education debt significantly boosts development and enjoyment consumption, while its impact on subsistence consumption is relatively limited. Based on this analysis, we propose Hypothesis 1:

H1: Households’ decision to incur education debt can effectively promote the growth of their total consumption expenditures, especially in the development and enjoyment consumption of households, while the impact on the subsistence consumption to meet basic needs is relatively limited.

From equation (11), the magnitude of a household’s education debt positively impacts its total consumption expenditure. Although increased education debt may temporarily strain disposable income due to repayment obligations, it is expected to significantly enhance household wealth and consumption capacity in the long term by boosting members’ future income potential. As education debt rises, households may reallocate their consumption structure, reducing non-essential spending to prioritize education-related expenditures. This shift reflects a transition from basic survival to development and enjoyment consumption, highlighting the high value placed on education and personal development.

Theoretically, subsistence consumption is essential and rigid, with households prioritizing basic needs even under financial constraints. Thus, education debt has limited impact on it. In contrast, development and enjoyment consumption is more flexible. Households see education debt as a long-term investment with positive returns, encouraging them to invest in education and shift towards these types of consumption. This trend highlights the importance of human capital investment and signals an upgrade in household consumption patterns.

Based on this analysis, we propose Hypothesis 2:

H2: The expansion of the scale of education debt can significantly boost household consumption, particularly in development and enjoyment consumption, while having limited impact on subsistence consumption.

Consumption upgrading refers to both the improvement of households’ consumption levels and the optimization of their consumption structure [38]. As China’s industrial structure evolves and the consumption environment improves, basic needs are increasingly met, giving rise to more personalized and diversified consumption demands [39]. This shift directly drives the improvement of overall consumption levels. In addition, according to Maslow’s Hierarchy of Needs theory, when the basic physiological and safety needs are satisfied, people will pursue higher-level needs, such as emotion, respect and self-actualization, and this upgrading of needs is reflected in consumption behavior by the increased proportion of development and enjoyment consumption in total consumption [40]. Therefore, this paper will use the ratio of development and enjoyment consumption to total household consumption $\frac{C_{e,t}+E_{t}}{C_{s,t}+C_{e,t}+E_{t}}$ as an indicator of the degree of household consumption upgrading. Given that education debt mainly affects development and enjoyment consumption, with the increase of$\;D_{E}$, household development and enjoyment consumption$\;\left(C_{e,t}+E_{t}\right)$increases, further contributing to the ratio $\frac{C_{e,t}+E_{t}}{C_{s,t}+C_{e,t}+E_{t}}$. Based on this, we propose Hypothesis 3:

H3: Education debt will have a positive impact on the upgrading of household consumption structure.

### 3.2 Consideration of investment decisions

Based on the precautionary savings theory, households usually choose to increase their precautionary savings in order to cope with potential future uncertainties, and ensure that they have sufficient funds to cope in case of an emergency [41]. However, risk-preference households exhibit a different pattern of behavior. Such households are more inclined to cut back on the proportion of precautionary savings and instead invest this amount in financial assets, willingly taking on higher risks in pursuit of potentially higher returns [42]. In view of this, this paper further incorporates investment decisions into the analytical framework of the intertemporal consumption model to explore how household risk preferences play a moderating role between education debt and household consumption.

It is assumed that the share of income that households spend on investment in period t is $\delta_{t}$. The larger the value $\delta_{t}$ taken, the more inclined the household is to make investment decisions, the higher the share of income it spends on investment, and the more risk-preferred it is.

At this point, the household is constrained by


$$\begin{array}{c} \begin{array}{c} W_{t+\text{1}}=(\text{1}+r)\left[W_{t}+\left(\text{1}-\delta_{t}\right)Y_{t}\cdot f\left(E_{t-\text{1}}\right)-C_{t}\right]+\;\left(\text{1}+i_{t}\right)\delta_{t}\;Y_{t}\cdot f\left(E_{t-\text{1}}\right)\\ \;\;\;\;\;\;\;\;=(\text{1}+r)\left[W_{t}+\left(\text{1}-\delta_{t}\right)Y_{t}\cdot f\left(E_{t-\text{1}}\right)-C_{s,t}-C_{e,t}-E_{t}\right]+\;\left(\text{1}+i_{t}\right)\delta_{t}\;Y_{t}\cdot f\left(E_{t-\text{1}}\right) \end{array} \end{array}$$ (12)

Where $\;\delta_{t}\;Y_{t}\cdot f\left(E_{t-\text{1}}\right)$ denotes the amount invested by the household in t period; $i_{t}$ denotes the return on investment (ROI) in period t, $i_{t}\geq -\text{1}$ is satisfied.

Next, consistent with the logic above, by constructing the Lagrangian function and solving it using the Euler equation, it is obtained:


$$\begin{array}{c} U^{\prime }\left(C_{t}\right)=\beta (\text{1}+r)U^{\prime }\left(C_{t+\text{1}}\right) \end{array}$$ (13)

$$\begin{array}{c} U^{\prime }\left(C_{t}\right)=\beta {\frac{\left(\text{1}+r)+\delta (i_{t}-r\right)}{\text{1}+r}Y}_{t+\text{1}}\frac{df\left(E_{t}\right)}{dE_{t}}U^{\prime }\left(C_{t+\text{1}}\right) \end{array}$$ (14)

According to equations (13) and (14), f(E) can be expressed as:


$$\begin{array}{c} f(E)=(\text{1}+r)\sqrt{\frac{\text{2}E}{\left[(\text{1}+r)+\delta \left(i_{t}-r\right)\right]Y_{t}}}+\varepsilon_{\text{2}} \end{array}$$ (15)

Where $\varepsilon_{\text{2}}$ is a constant term.

Substituting the equation $D_{E}=\alpha E$ and equation (15) into constraint (12), the total consumption expenditure $C_{t}$ of the household in period t is found to be:


$$\begin{array}{c} C_{t}=\sqrt{\text{2}\left[(\text{1}+r)+\delta \left(i_{t}-r\right)\right]Y_{t}E}+\varepsilon_{\text{2}}{\left[(\text{1}+r)+\delta \left(i_{t}-r\right)\right]Y}_{t}+W_{t}-\frac{W_{t+\text{1}}}{\text{1}+r}\\ \;=\sqrt{\frac{\text{2}\left[(\text{1}+r)+\delta \left(i_{t}-r\right)\right]Y_{t}D_{E}}{\alpha }}+\varepsilon_{\text{2}}{\left[(\text{1}+r)+\delta \left(i_{t}-r\right)\right]Y}_{t}+W_{t}-\frac{W_{t+\text{1}}}{\text{1}+r} \end{array}$$ (16)

The partial derivatives are obtained for education expenditure E and education debt${\;D}_{E}$, respectively:


$$\begin{array}{c} \frac{{\partial C}_{t}}{\partial E}=\sqrt{\frac{\left[(\text{1}+r)+\delta \left(i_{t}-r\right)\right]Y_{t}}{\text{2}E}}>\text{0} \end{array}$$ (17)

$$\begin{array}{c} \frac{{\partial C}_{t}}{\partial D_{E}}=\sqrt{\frac{\left[(\text{1}+r)+\delta \left(i_{t}-r\right)\right]Y_{t}}{\text{2}\alpha D_{E}}}>\text{0} \end{array}$$ (18)

Equations (17) and (18) show that both household education expenditure and education debt positively contribute to total household consumption expenditure. Given the rigidity of subsistence consumption, the impact of education debt on household consumption is primarily reflected in the growth of enjoyment consumption, thereby driving the upgrading and transformation of household consumption structure. That is, the ratio of development and enjoyment consumption to total household consumption $\frac{C_{e,t}+E_{t}}{C_{s,t}+C_{e,t}+E_{t}}$ is increased. Additionally, the investment ratio δ, which indirectly reflects household risk attitudes, and the return on investment (ROI)${\;i}_{t}\;$ are not fixed, suggesting that household risk preferences may moderate the positive relationship between education debt $D_{E}$ and consumption upgrading $\frac{C_{e,t}+E_{t}}{C_{s,t}+C_{e,t}+E_{t}}$. The direction of this moderating effect is uncertain and requires further exploration and verification through empirical analysis. Based on this, we propose Hypothesis 4:

H4: Household risk attitude plays a certain moderating role between education debt and consumption structure upgrading.

## 4. Research design

### 4.1 Sample selection and data sources

The data used in this study are from the China Household Finance Survey (CHFS) conducted by the China Household Finance Survey and Research Center at Southwestern University of Finance and Economics (SWUFE). The survey spans five periods from 2013 to 2021 and covers 29 provinces, autonomous regions, and municipalities, excluding Xinjiang, Tibet, and Hong Kong, Macao, and Taiwan. It comprehensively collects information on household assets, debts, demographics, consumption, and income, providing high-quality micro-data for research on China’s household finance. To ensure the reliability of our findings, we processed the data as follows: Firstly, we deleted household observations with data missing values (In the CHFS dataset, there are data entries such as “.d”, “.r”, “.e”, and “.n”. Specifically, “.d” indicates that the respondent did not know how to answer, resulting in missing data; “.r” indicates that the respondent refused to answer the question, resulting in missing data; “.e” indicates that the respondent was not asked the question, resulting in missing data; “.n” indicates that during data verification, it was found that the respondent did not provide an answer to a particular question, but instead the interviewer made a speculative response (i.e., guessed the answer), and this speculative answer was not included in the database, resulting in missing data.). And according to the Civil Code of the People’s Republic of China, a natural person aged 18 or above is considered an adult with full capacity for civil conduct. Therefore, we excluded household samples where the head of the household was under 18 years of age. Secondly, when completing the China Household Finance Survey questionnaires, household members may intentionally conceal their true financial situations, leading to discrepancies between the reported data and actual circumstances. Such misreporting generates anomalous values in the dataset (e.g., owning 9,999 houses, negative income, or negative expenditures), so we log-transformed households’ consumption expenditures, income, assets, and debts and perform a 1% upper and lower tail indentation on all continuous variables. Finally, we extracted relevant household data, calculated the main independent variable of education debt, and categorized total consumption expenditure into subsistence, development, and enjoyment consumption based on prior theoretical analyses, resulting in a final sample of 100,608 observations.

### 4.2 Modeling

Households with education debt, influenced by time preferences and subjective decision-making factors, face different budget constraints compared to those without such debts [43]. Their consumption expenditures and structures may thus differ from households without education debt. Therefore, whether a household has education debt and the scale of these debts can have varying impacts on total consumption and consumption structure. Based on the above analysis, this paper constructs the following empirical model:


$$\begin{array}{c} \text{ln}\;C_{it}=\alpha_{\text{0}}+\alpha_{\text{1}}deb{t\_if}_{it}+\alpha_{\text{2}}I_{it}+\alpha_{\text{3}}F_{it}+Y_{t}+\lambda_{i}+\in_{it} \end{array}$$ (19)

$$\begin{array}{c} \text{ln}\;C_{it}=\beta_{\text{0}}+\beta_{\text{1}}{lndebt\_edu}_{it}+\beta_{\text{2}}I_{it}+\beta_{\text{3}}F_{it}+Y_{t}+\lambda_{i}+\in_{it} \end{array}$$ (20)

Where the subscript i denotes the household, and t denotes the year. The dependent variable $lnC_{it}$ is the logarithm of total household consumption expenditure, household subsistence consumption expenditure, and development and enjoyment consumption expenditure, i.e., indicating the level of household consumption in the year. $deb{t\_if}_{it}$ is the independent variable, a dummy variable indicating whether the household has education debt. ${lndebt\_edu}_{it}$ is the logarithm of the household’s education debt. $I_{it}$ denotes a set of control variables at the head of household level, $F_{it}$ denotes a set of control variables at the household level. The subscripts$\;Y_{t}\;$, $\lambda_{i}$ and $\in_{it}$ represent year fixed effects, household fixed effects and the error term, respectively.

Finally, a benchmark model is constructed to verify the impact of education debt on household consumption upgrading:


$$\begin{array}{c} upgrade_{C_{it}}=\sigma_{\text{0}}+\sigma_{\text{1}}{lndebt\_edu}_{it}+\sigma_{\text{2}}I_{it}+\sigma_{\text{3}}F_{it}+Y_{t}+\lambda_{i}+\in_{it} \end{array}$$ (21)

Where $upgrade_{C_{it}}$ is the consumption upgrading variable of the household, and the other variables are consistent with the implications of the above model. Moreover, in contrast to Model (20), which focuses on analyzing the absolute level of household consumption, Model (21) captures the relative changes in the optimization of household consumption structure, directly reflecting the qualitative shift from subsistence to development and enjoyment consumption. The two models complement each other, forming an effective and synergistic analytical framework.

### 4.3 Definition and measurement of key variables

#### 4.3.1 Dependent variables: total household consumption, subsistence consumption, development and enjoyment consumption, degree of consumption upgrading.

This study examines the relationship between household education debt and consumption upgrading by analyzing its impact on total household consumption and consumption structure. For total household consumption, we use the logarithm of total household consumption expenditures, which includes eight subcategories: food, clothing, housing, household equipment and services, transportation and communication, education, recreation and entertainment, healthcare, and other consumption. For consumption structure, we categorize expenditures into two types [44]: subsistence consumption (basic living needs) and development and enjoyment consumption (higher-level needs such as education, culture, and recreation). Additionally, we measure the degree of consumption upgrading using the ratio of development and enjoyment consumption to total household consumption [45].

#### 4.3.2 Core independent variables: education debt decision, education debt scale.

The independent variables of this study are education debt decision and education debt scale. Among them, the presence or absence of education debt is used as a dummy variable, assigning a value of 1 to households with education debt and 0 to households without education debt; the size of education debt is expressed as the logarithm of the household’s total education debt (${lndebt\_edu}_{it}$). And detailed descriptive statistics of education debt were conducted in S1 Appendix.

#### 4.3.3 Control variables selection and measurement.

Building on prior research, this study controls for other factors influencing household consumption by selecting two levels of control variables: the household head level and the household level [46]. At the household head level, control variables include gender (1 for male, 0 for female), age, age squared divided by 100, years of education(coded based on highest education level), marital status(1 for married, 0 for unmarried), health status(1 for very good/good, 0 for average/bad), and party membership(1 for member, 0 for non-member) [47–51]. At the household level, control variables include logarithm of household income, logarithm of housing debts, household size, logarithm of total household assets, child dependency ratio(percentage of minor children), elderly dependency ratio(percentage of household members aged 60+), and household employment rate(ratio of employed individuals to total household members) [52–58]. These variables account for various internal and external factors that may affect household consumption expenditure, yielding more precise results. Table 1 presents the descriptive statistics of the relevant variables after data cleaning. Meanwhile, the relevant descriptive statistical tables of the data cleaning process are placed in S2 Appendix.

**Table 1:** Descriptive statistics.

Variable	Households with education debt (Sample: 3039)				Households without education debt (Sample: 97569)			
	Max	Min	Mean	Std. Dev	Max	Min	Mean	Std. Dev
Total household consumption	373759.9	4618	59997.5	52836.6	373759.9	4618	65831.8	62707.5
Total household consumption(ln)	12.8313	8.4379	10.7207	0.7501	12.8313	8.4379	10.7337	0.8722
Subsistence consumption	179300	2050	26361.8	23761.8	179300	2050	32220.8	28621.6
Subsistence consumption(ln)	12.0968	7.6261	9.8608	0.8134	12.0968	7.6260	10.0482	0.8533
Development and enjoyment consumption	256000	760	33205.5	36990.9	256000	760	32879.7	41714.8
Development and enjoyment consumption(ln)	12.4529	6.6340	9.9741	0.9653	12.4529	6.6346	9.7950	1.1591
Consumption upgrading	1	0.0184	0.5244	0.2027	1	0.00	0.4484	0.2035
Total education debt	20000	0.25	12949.2	7103.9	0	0	0	0
Total education debt(ln)	9.9035	0.2231	9.1692	1.0200	0	0	0	0
**Head of the household level**								
Gender dummy	1	0	0.8163	0.3872	1	0	0.7668	0.4228
Age	100	18	48.9480	9.4378	112	18	53.6578	13.6423
Age^2^/100	100	3.24	24.8495	9.9115	125.44	3.24	30.6527	14.8432
Year of education	22	0	8.4945	3.3700	22	0	9.4606	3.9988
Marital dummy	1	0	0.8953	0.3061	1	0	0.8688	0.3375
Health dummy	1	0	0.6416	0.4795	1	0	0.7639	0.4246
Party member dummy	1	0	0.2823	0.4502	1	0	0.3652	0.4815
**Household level**								
Household income(ln)	13.3068	0	9.7721	2.1916	13.3068	0	10.3794	2.0359
Household size	19	1	4.3695	1.7426	21	1	3.4218	1.7438
Household assets(ln)	16.0670	7.6829	11.7847	1.5293	16.0670	7.6829	12.7330	1.6920
Housing debts(ln)	13.3573	0	2.6229	4.6418	13.3573	0	1.5909	3.9802
Child dependency ratio	0.5	0	0.1702	0.1750	0.5	0	0.1296	0.1672
Elderly dependency ratio	1	0	0.1015	0.1806	1	0	0.2983	0.3854
Household employment rate	1	0	0.4822	0.2525	1	0	0.4712	0.3316

To better illustrate the consumption patterns of households with and without education debts, this study divides the sample into two groups and analyzes their expenditure characteristics. The descriptive statistics show that households with education debt exhibit slightly lower average total consumption levels compared to those without such debt. Notably, households with education debt demonstrate a relatively smaller standard deviation in consumption. Since a larger standard deviation indicates greater data dispersion, these results suggest that consumption patterns are more concentrated among households with education debt. Additionally, while households with education debt have lower average subsistence consumption, their development and enjoyment consumption are higher than those of households without education debt. This suggests that education debt may shift consumption towards more flexible, higher-level needs, facilitating household consumption upgrading.

Regarding household head characteristics, those with education debt are youngers and predominantly married, indicating that younger households may prioritize education’s impact on future careers and social status. The lower educational attainment likely reflects an earlier entry into the labor market to alleviate pressure from children’s education debt.

In terms of household characteristics, those with education debt have lower assets and income, possibly due to financial constraints that limit investments in income-generating activities. Conversely, higher housing debts suggest that these households may have better access to credit through established relationships with financial institutions, facilitating education loan applications.

## 5. Empirical results and analysis

### 5.1 Baseline regression

#### 5.1.1 Analysis of the impact of Households’ education debt decisions on consumption expenditures.

Table 2 presents the baseline regression results on the impact of education debt on household consumption upgrading, controlling for household head characteristics, household fixed effects and year fixed effects. Columns (1), (4), and (7) introduce the education debt dummy variable, while columns (2), (5), and (8) progressively add control variables for household head and household characteristics to mitigate omitted variable bias and verify regression robustness. The results show that households with education debt have total consumption, development and enjoyment consumption levels 13.13% and 25.24% higher, respectively, than those without education debt while the regression coefficient for promoting subsistence consumption is relatively small. This suggests limited impact on basic survival needs and indicates that education debt alleviates liquidity constraints, enabling households to pursue higher-level consumption and optimize their consumption structure. Children’s education investment is categorized under development and enjoyment consumption, as education is a developmental and high-quality form of consumption. With rising education costs, the share of development and enjoyment consumption in total consumption increases. This confirms that education debt not only boosts overall household consumption, but also promotes consumption structure upgrading. Thus, Research Hypothesis 1 is supported.

**Table 2:** Benchmark regression results on the impact of households’ education debt decisions on consumption expenditures.

Variable	Total household consumption(ln)			Subsistence consumption(ln)			Development and enjoyment consumption(ln)		
	(1)	(2)	(3)	(4)	(5)	(6)	(7)	(8)	(9)
Education debt dummy	0.1353***	0.1329***	0.1313***	0.0298*	0.0304*	0.0314*	0.2593***	0.2537***	0.2524***
	(8.5423)	(8.4532)	(8.6793)	(1.7131)	(1.7624)	(1.8682)	(11.9314)	(11.7376)	(12.0373)
Gender dummy		0.0386***	0.0139*		0.0394***	0.0201**		0.0389***	0.0054
		(4.6874)	(1.7713)		(4.7490)	(2.4877)		(3.1957)	(0.4617)
Age		−0.0000	−0.0089***		−0.0011	−0.0080***		0.0013	−0.0112***
		(−0.0035)	(−4.3293)		(−0.4987)	(−3.6902)		(0.4077)	(−3.7420)
Age^2^/100		−0.0062***	0.0049**		−0.0033	0.0053***		−0.0104***	0.0053*
		(−2.9529)	(2.5466)		(−1.5711)	(2.6292)		(−3.3679)	(1.8854)
Year of education		0.0106***	0.0086***		0.0090***	0.0073***		0.0134***	0.0107***
		(6.8239)	(5.8763)		(5.7237)	(4.8119)		(5.8978)	(4.9952)
Marital dummy		0.1501***	0.0612***		0.1582***	0.0897***		0.1682***	0.0477***
		(11.9345)	(5.1932)		(12.6811)	(7.4889)		(9.0937)	(2.7228)
Health dummy		0.0091	−0.0094		0.0485***	0.0329***		−0.0276***	−0.0525***
		(1.2811)	(−1.3896)		(6.6953)	(4.6666)		(−2.7136)	(−5.3658)
Party member dummy		−0.0346***	−0.0093*		−0.0238***	−0.0041		−0.0418***	−0.0065
		(−6.7951)	(−1.8995)		(−4.5417)	(−0.7953)		(−5.6001)	(−0.8950)
Household income(ln)			0.0339***			0.0255***			0.0437***
			(23.6725)			(17.1875)			(21.1800)
Household size			0.0783***			0.0589***			0.1070***
			(33.5043)			(23.5420)			(32.6673)
Household assets(ln)			0.0906***			0.0733***			0.1232***
			(36.0127)			(28.2115)			(32.9924)
Housing debts(ln)			0.0045***			0.0050***			0.0012
			(6.3046)			(6.3502)			(1.2282)
Child dependency ratio			0.1096***			0.1445***			0.1715***
			(4.0888)			(4.9794)			(4.5523)
Elderly dependency ratio			−0.1179***			−0.0744***			−0.2035***
			(−8.0871)			(−4.9754)			(−9.3132)
Household employment rate			−0.0119			0.0400***			−0.0432***
			(−1.1176)			(3.6085)			(−2.7490)
Household fixed effects	yes	yes	yes	yes	yes	yes	yes	yes	yes
Year fixed effects	yes	yes	yes	yes	yes	yes	yes	yes	yes
Observations	100,608	100,608	100,608	100,608	100,608	100,608	100,608	100,608	100,608
R-squared	0.7356	0.7392	0.7621	0.7087	0.7118	0.7264	0.6839	0.6873	0.7112

Notes: Numbers in parentheses are heteroskedasticity-consistent standard errors, clustered at the household level. ***, **, and * indicate that the parameter estimate is significantly different from zero at the 1%, 5%, and 10% level, respectively.

*** p < 0.01, ** p < 0.05, * p < 0.1

#### 5.1.2. Analysis of the impact of the size of the households’ education debt on consumption expenditures.

The previous section examined how education debt affects household consumption expenditure, finding that households with such debt generally have higher consumption propensities than those without debt. However, does a larger scale of education debt necessarily lead to greater consumption expenditure? Table 3 reports the effects of the education debt scale on household consumption expenditure. The regression results show that the scale of education debt positively correlates with total household consumption, development and enjoyment consumption, indicating that moderate increases in education debt significantly boost these expenditures. In contrast, although the impact on subsistence consumption is significantly positive, the extent of the influence is not obvious enough, likely due to its relatively stable nature as a core household expenditure. Thus, increasing education debt can enhance household wealth and purchasing power over time, particularly benefiting development and enjoyment consumption. This supports Hypothesis 2.

**Table 3:** Benchmark regression results on the impact of households’ education debt scale on consumption expenditures.

Variable	Total household consumption(ln)			Subsistence consumption(ln)			Development and enjoyment consumption(ln)		
	(1)	(2)	(3)	(4)	(5)	(6)	(7)	(8)	(9)
Total education debt(ln)	0.0153***	0.0149***	0.0148***	0.0032*	0.0032*	0.0034*	0.0292***	0.0286***	0.0285***
	(8.8609)	(8.7346)	(9.0322)	(1.6990)	(1.7188)	(1.8648)	(12.3277)	(12.0994)	(12.4756)
Head of household control variables	No	Yes	Yes	No	Yes	Yes	No	Yes	Yes
Household control variables	No	No	Yes	No	No	Yes	No	No	Yes
Household fixed effects	Yes	Yes	Yes	Yes	Yes	Yes	Yes	Yes	Yes
Year fixed effects	Yes	Yes	Yes	Yes	Yes	Yes	Yes	Yes	Yes
Observations	100,608	100,608	100,608	100,608	100,608	100,608	100,608	100,608	100,608
R-squared	0.7356	0.7392	0.7622	0.7087	0.7118	0.7264	0.6840	0.6874	0.7113

Note: The control variables include two parts: the household head characteristic variable and the household characteristic variable, which are specifically described in the third section of the fourth part of the article. The following table is the same.

#### 5.1.3 Analysis of the impact of households’ education debt on consumption upgrading.

Table 4 presents the benchmark regression results on the impact of education debt on household consumption upgrading, controlling for household head characteristics, household fixed effects and year fixed effects. The results indicate that education debt significantly and positively affects household consumption upgrading at the 1% level, with higher education debt associated with an increased share of development and enjoyment consumption in total household consumption. This suggests a growing trend of consumption upgrading among residents. From the scatter diagram and fitted line of the relationship between education debt and household consumption upgrading in S2 Fig, it is not difficult to see that there is indeed a relatively clear positive linear relationship between them. Regarding control variables, increased household income directly enhances purchasing power, allowing households to buy more goods and services, including non-essential and luxury items, thereby promoting consumption expenditure and upgrading. Higher household assets may also create a wealth effect, making households feel more financially secure and willing to consume and invest in quality-of-life improvements. Additionally, a larger household size may increase total income through more labor force participation and improve consumption efficiency through labor division and sharing. Zhang et al. (2019) found that household debt, particularly housing debt, has a more significant inhibitory effect on consumption expenditures and consumption upgrading among Chinese households [59]. To address potential endogeneity issues from omitted variables, this study controls for household housing debt, which also shows a negative impact on consumption upgrading. Overall, the results confirm that education debt promotes household consumption upgrading, supporting Research Hypothesis 3.

**Table 4:** Benchmark regression results on the impact of households’ education debt on consumption upgrading.

Variable	Consumption upgrading		
	(1)	(2)	(3)
Total education debt(ln)	0.0055***	0.0054***	0.0053***
	(10.2474)	(10.0000)	(9.8914)
Head of household control variables	No	Yes	Yes
Household control variables	No	No	Yes
Household fixed effects	Yes	Yes	Yes
Year fixed effects	Yes	Yes	Yes
Observations	100,608	100,608	100,608
R-squared	0.5103	0.5114	0.5173

### 5.2 Robustness tests

#### 5.2.1 PSM estimation.

**Fig 1:**
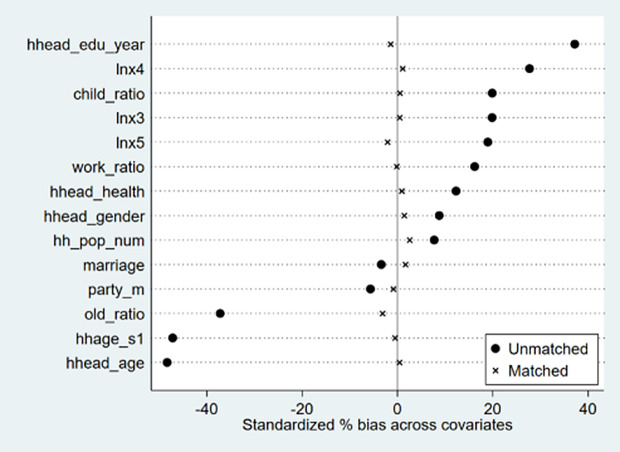
Covariate imbalance between group means before and after matching.

To ensure the robustness of the research conclusions, this study employs Propensity Score Matching (PSM) to re-estimate the treatment effects. This method effectively alleviates potential self-selection bias by constructing highly matched treatment and control group samples based on observable characteristics. Specifically, we divide the household samples into two groups according to whether households are averse to risk: Group 1 (treatment group, risk-averse household) and Group 2 (control group, risk-preference household). Under the control of household fixed effects and year fixed effects, we use the involved control variables as PSM covariates. After re-matching the samples using nearest neighbor matching (1:4) and conducting regression analysis, we intuitively assess the improvement in sample balance between groups by presenting the test results of covariate group deviations in Fig 1 and the balance test result is described in more detail in S3 Appendix. These results show that after Propensity Score Matching (PSM), the standardized deviations of all covariates are significantly reduced, indicating a noticeable improvement in sample balance. This balance test result verifies the effectiveness of the matching method. Column (1) of Table 5 reports the regression analysis results of the matched samples. The study finds that education debt has a significant promoting effect on household consumption upgrading, which is statistically significant at the 1% significance level.

**Table 5:** Regression results of robustness tests.

Variable	PSM estimation	Replacement of the independent variable	Replacement of the dependent variable	Narrowing the sample
	(1)	(2)	(3)	(4)
Total education debt(ln)	0.0063***			0.0054***
	(5.1094)			(9.1122)
Education debt-to-asset ratio (ln)		0.0374***		
		(3.7704)		
Structural coefficient of consumption upgrading			0.0053***	
			(9.8914)	
Head of household control variables	Yes	Yes	Yes	Yes
Household control variables	Yes	Yes	Yes	Yes
Household fixed effects	Yes	Yes	Yes	Yes
Year fixed effects	Yes	Yes	Yes	Yes
Observations	21,432	100,554	100,608	64,851
R-squared	0.5960	0.5168	0.5173	0.5356

#### 5.2.2 Replacement of the independent variable.

In the baseline regression, we utilize the absolute value of education debt as the core explanatory variable. To further validate the robustness of our findings, this study draws upon the methodology of Campbell and Cocco (2007) by employing a relative metric normalized by household asset size—education debt-to-asset ratio. We apply logarithmic transformation to this ratio to address heteroskedasticity concerns and enhance model performance before conducting re-estimation. This relative measure offers two principal advantages: first, it substantially improves cross-sectional comparability across samples by eliminating scale effects attributable to household size variations; second, it provides a more precise reflection of households’ effective education debt burden.

#### 5.2.3 Replacement of the dependent variable.

Drawing on Xu and Jiang’s (2015) method for quantifying industrial upgrading, we construct a consumption upgrading indicator to examine how household debt affects the structural shift in household consumption [60]. Specifically, we separately weight subsistence consumption, development and enjoyment consumption in our calculations:


$$\begin{array}{c} Cons\_update=\text{1}\cdot C_{s}+\;\text{2}\cdot C_{f} \end{array}$$ (22)

Among them, Cons_update indicates the coefficient of consumption upgrading structure, $C_{s}$ indicates the proportion of subsistence consumption in total household consumption, and $C_{f}$ indicates the proportion of development and enjoyment consumption in total household consumption. Higher consumption tiers are assigned greater weights, yielding larger coefficients that more prominently reflect household consumption upgrading. And the test results are shown in the third column of Table 5, and the significance of the variables has not changed compared to before. It still indicates that households with education debt have higher demand for consumption upgrading goods and services, which helps to promote consumption upgrading.

#### 5.2.4 Narrowing the sample.

To further test the robustness of the regression results, the sample is narrowed down to a sample of household heads aged 18–60 for the regression in order to mitigate the possible bias due to the differences in the impact of the age of the head of the household on the upgrading of household consumption. The fourth column of Table 5 reports the results of the robustness test, where the coefficient on the independent variable of the regression results after narrowing the sample is still significantly positive, and this coefficient has a significance level of 1% in a statistically significant way. In addition, the direction and size of the coefficients of the control variables are basically not significantly different, so it can be seen from the above that education debt has a substantial promotion effect on household consumption upgrading.

#### 5.2.5 Two-stage regression (2SLS) test using instrumental variable method.

Education debt can affect the consumption upgrading, but households with a stronger preference for development and enjoyment consumption may also incur education debt, making it difficult to identify the causal relationship between education debt and the consumption upgrading. Therefore, in this paper, the logarithm of the mean education debt of other households in the community where the household is located, except for this household, is selected as the instrumental variable to conduct a two-stage least squares (2SLS) regression analysis, with the aim of effectively alleviating the endogenous interference caused by reverse causality.

The empirical results are shown in Table 6, among which column (1) reports the first-stage regression results and F-values of 2SLS. Column (2) reports the regression results of the second stage of 2SLS and the F value of the Cragg-Donald Wald test. Firstly, the F-value of the first stage of the 2SLS regression results is 339.73, and the F-value of the second stage Cragg-Donald Wald test is 663.03. Both are significantly greater than the critical value of the 10% bias level. Therefore, the null hypothesis of weak instrumental variables is rejected. Secondly, from the first-stage regression results of 2SLS, it can be known that the selected instrumental variable has a certain correlation with the education debt of this household, thus meeting the endogeneity condition of the instrumental variable. Finally, the second-stage regression results of 2SLS show that when the explained variable is consumption upgrade, the regression coefficient of education debt is significantly positive. It once again confirms that household education debt in our country will significantly promote consumption upgrading.

**Table 6:** The 2SLS regression results of households’ education debt and consumption upgrading.

Variable	(1)	(2)
	Total education debt(ln)	Consumption upgrading
Total education debt(ln)		0.0136***
		(0.0049)
Average education debt of other households in the community (ln)	−0.0507***	
	(0.0028)	
Head of household control variables	Yes	Yes
Household control variables	Yes	Yes
Household fixed effects	Yes	Yes
Year fixed effects	Yes	Yes
Observations	100506	100506
R-squared		0.058
Adj. R-squared		0.041
The F value of the first stage	339.73	
Cragg-Donald Wald		663.03

This indicates that, when considering the endogeneity issue of reverse causality, the empirical analysis results of this paper remain reliable and the inferences still hold.

## 6. Further analysis

### 6.1 Mechanism analysis

#### 6.1.1 Analysis of online consumption’s mediating role.

China has entered the “Education + Internet” era, where increasing costs of “Education + Internet” consumption reflect rising household demand for educational resources [61]. The Internet provides a cost-effective way to meet these needs through diverse resources such as online courses and educational software, significantly boosting household development and enjoyment consumption [62]. There are t wo main reasons why education debt promotes Internet consumption. First, Chinese households place a high emphasis on educational investment, with increased education debt reflecting their expectations of improving their children’s education [63]. Second, the Internet offers abundant information, allowing households to compare the quality and prices of educational products and services [64]. This enables them to make more informed choices, increasing the efficiency of education debt and facilitating easier access to and management of education loans. Additionally, the rise of online lending platforms and mobile payment tools has enhanced financial services, providing households with more consumption channels and credit resources [65]. This not only promotes consumption upgrading but also influences how households manage education debt.

In summary, education debt may promote household Internet consumption through various pathways, with this relationship potentially deepening as Internet technology continues to evolve. To further analyze whether education debt drives household consumption structure upgrading via increased Internet consumption, this study draws on and employs Internet consumption as a mediating variable. Following the stepwise testing procedure for mediation analysis, we establish the following three econometric models:


$$\begin{array}{c} UC_{it}=\gamma_{\text{1}}+{alndebt_{edu}}_{it}+\sum_{i=\text{1}}^{N}\theta_{i}X_{it}+Y_{t}+\lambda_{i}+\in_{it} \end{array}$$ (23)

$$\begin{array}{c} M_{it}=\gamma_{\text{2}}+b{lndebt_{edu}}_{it}+\sum_{i=\text{1}}^{N}\eta_{i}X_{it}+Y_{t}+\lambda_{i}+\in_{it} \end{array}$$ (24)

$$\begin{array}{c} UC_{it}=\gamma_{\text{1}}^{\prime }+{a^{\prime }lndebt_{edu}}_{it}+cM_{it}+\sum_{i=\text{1}}^{N}\partial_{i}X_{it}+Y_{t}+\lambda_{i}+\in_{it} \end{array}$$ (25)

In the model, the dependent variable$\;UC_{it}\;$is the ratio of household development and enjoyment consumption to total household consumption, serving as a proxy for consumption upgrading. The mediating variable$\;M_{it}\;$represents the Internet consumption level of households in period t, and $X_{it}$ represents the control variables for household i in period t.

As shown in Table 7, education debt has a significantly positive effect on household Internet consumption, with a promotion effect of approximately 2.16% (column 1). In the test of Model (25), the regression coefficients of $M_{it}$ and ${lndebt_{edu}}_{it}$ are all significantly positive (column 2). It indicates that the Internet consumption level of households has played a partial mediating role between education debt and consumption upgrading. The P-value of the Sobel test is 0.012, further verifying the mediating effect of the Internet consumption level of households.

**Table 7:** Regression results for the mediating effect of Internet consumption level.

Variable	(1)	(2)
	Internet consumption(ln)	Consumption upgrading
Total education debt(ln)	0.0216***	0.0053***
	(2.9238)	(9.7861)
Internet consumption(ln)		0.0026***
		(9.9501)
Head of household control variables	Yes	Yes
Household control variables	Yes	Yes
Household fixed effects	Yes	Yes
Year fixed effects	Yes	Yes
Observations	100,608	100,608
R-squared	0.6969	0.5180

As households increasingly engage in online consumption activities, this new consumption pattern has significantly transformed both the structure and level of household expenditure, making the mediating effect of Internet consumption a critical factor that cannot be overlooked. Internet consumption not only offers households greater choice and convenience but also efficiently meets their developmental and enjoyment-oriented needs [66]. By supplementing and partially replacing offline consumption, it drives the upgrading of household consumption structures. Additionally, Internet consumption can both directly influence household consumption structures and indirectly promote upgrading by shaping consumption habits and expectations [67]. As noted by Zeng and Zhong (2021), Internet use positively correlates with consumption upgrading by providing more information, increasing choices, and lowering transaction costs [68]. This suggests that Internet consumption significantly impacts the upgrading of household consumption. Therefore, as education debt expands, household may increasingly adopt Internet consumption, optimizing and upgrading their consumption structures. This supports the notion that Internet consumption is a key mediating pathway through which education debt drives consumption upgrading.

#### 6.1.2 Analysis of the moderating effect of risk attitudes.

Risk attitudes can influence how household education debt affects consumption upgrading [69]. Risk-preference households may reduce this effect for two main reasons.

On the one hand, regarding the relationship between risk preferences and consumption behavior, risk-preference households are generally more willing to engage in high-risk investments in pursuit of higher returns [70]. Such households tend to prioritize asset appreciation over stable consumption expenditures. Consequently, they may be less inclined to use debt to smooth consumption and instead prefer to allocate funds to investment channels that potentially yield higher returns. This investment behavior may reduce household liquidity available for consumption, thereby weakening the role of education debt in promoting household consumption upgrading. On the other hand, in terms of household economic vulnerability, risk-preference households may face greater financial fragility, as their investment decisions could lead to increased income volatility [71]. Under economic pressure, such households might cut back on non-essential consumption, including education-related expenses, to maintain financial stability. As a result, the positive effect of education debt on consumption upgrading for these households could be further diminished. Also, we introduce an interaction term between education debt and risk attitude, with results presented in Table 8. In column (3), the interaction term’s coefficient is significantly negative, despite education debt positively affecting household consumption upgrading. This indicates that compared with risk-averse households, education debt in risk-preference households has a relatively smaller promoting effect on the upgrading of household consumption. Overall, risk-preference households may negatively moderate the impact of education debt on consumption upgrading due to factors such as a greater inclination toward risky investments and higher economic vulnerability. This confirms Hypothesis 4.

**Table 8:** Regression results of the moderating effect test for risk attitudes.

Variable	Risk-attitudes moderating effect		
	(1)	(2)	(3)
Total education debt(ln)	0.0108***	0.0091***	0.0093***
	(14.1266)	(11.8694)	(12.1650)
Risk attitudes	0.0170***	0.0113***	0.0088***
	(29.6020)	(19.1808)	(15.1149)
Total education debt × Risk attitudes	−0.0013***	−0.0009***	−0.0008**
	(−4.1455)	(−2.7475)	(−2.5421)
Head of household control variables	No	Yes	Yes
Household control variables	No	No	Yes
Household fixed effects	Yes	Yes	Yes
Year fixed effects	Yes	Yes	Yes
Observations	100,605	100,605	100,605
R-squared	0.1051	0.1261	0.1501

### 6.2 Heterogeneity analysis

As China’s education level rapidly develops, households are increasingly prioritizing education and personal development. This shift in mindset may drive higher household spending on education and related areas. However, different households may respond differently to education debt when seeking better employment opportunities, social status, and long-term economic and social rewards. For example, households with varying income levels, from urban and rural areas, and with different levels of financial literacy and debt levels may have distinct affordability and responses to education debts [72]. As a result, consumption patterns may exhibit diverse characteristics and trends.

#### 6.2.1 Heterogeneity of households in different income brackets.

According to the relevant studies on the important impact of household income on household consumption, all the samples in this paper are divided into three groups: low-income, middle-income and high-income, to examine the heterogeneous role of household income classes in the impact of education debt on consumption upgrading [73]. Among them, the top 20% of income-ranked households are high-income households, the bottom 20% are low-income households, and the remaining households are defined as middle-income households. The regression results are included in Table 9 columns (1) to (3), respectively. Table 9 shows that the positive impact of education debts on the consumption upgrading of middle- and low-income households is more significant. Specifically, the impact of education debt on consumption upgrading is significant at the 1% level for middle- and low-income families, while its impact on high-income households is only significant at the 10% level. This suggests that education debt has a more substantial effect on consumption upgrading for lower-income groups.

**Table 9:** Heterogeneity analysis based on households in different income brackets.

Variable	Consumption upgrading		
	Low-income	Middle-income	High-income
	(1)	(2)	(3)
Total education debt (ln)	0.0061***	0.0054***	0.0040*
	(4.0834)	(7.3583)	(1.8849)
Head of household control variable	Yes	Yes	Yes
Household control variables	Yes	Yes	Yes
Household fixed effects	Yes	Yes	Yes
Year fixed effects	Yes	Yes	Yes
Observations	11,781	50,128	12,816
R-squared	0.5482	0.5466	0.5876

The reasons for these differences are as follows: Middle-income households, who are highly concerned about social status, are more likely to invest in education to enhance or maintain their social standing [74]. They often have sufficient resources to afford high-quality education, including cramming classes, training courses, and educational tours. Meanwhile, low-income households, influenced by national education policies, are becoming more aware of the value of education and adjusting their consumption structure to prioritize educational spending [75]. This shift promotes consumption upgrading even with limited resources. In contrast, high-income households may prefer to allocate their wealth to other forms of asset accumulation [76], such as real estate or stocks, rather than focusing on consumption upgrading. These households typically have greater financial resilience to manage education loans without compromising other aspects of household consumption. Overall, this paper argues that education debt plays a more significant role in consumption upgrading for middle- and low-income households compared to high-income households.

#### 6.2.2 Heterogeneity in the level of household financial literacy.

Household heads with higher financial literacy are more likely to recognize the long-term benefits of educational investment [77]. This understanding can shift household spending toward development and enjoyment consumption. These household heads may also be more proactive in using education loans to fund their children’s education while minimizing the impact on immediate spending through careful financial planning.

Second, household heads with higher financial literacy are better equipped to identify and use financial tools like education savings accounts and loans. This helps optimize funding sources for education, reducing financial pressure and freeing up funds for other consumption areas [78]. Additionally, they tend to make more rational and forward-looking consumption decisions, focusing not only on immediate satisfaction but also on long-term benefits and quality of life. As a result, they are more likely to invest in areas like education, a tendency supported by empirical studies, which can drive consumption upgrading.

In the current economic context, financial literacy significantly influences household consumption behaviors [79]. To explore this relationship, this study uses household heads’ accurate answers to three questions on compound interest, inflation, and investment risk from the 2015 CHFS survey to categorize financial literacy into four levels: low, medium, high, and very high. We then re-estimate the impact of education debt on household consumption upgrading across these groups, with results presented in Columns (1) to (4) of Table 10.

**Table 10:** Heterogeneity analysis based on household financial literacy.

Variable	Consumption upgrading			
	Low financial literacy	Medium financial literacy	High financial literacy	Very high financial literacy
	(1)	(2)	(3)	(4)
Total education debt(ln)	0.0044***	0.0056***	0.0065***	0.0084***
	(5.7320)	(5.4708)	(4.8932)	(3.6032)
Head of household control variables	Yes	Yes	Yes	Yes
Household control variables	Yes	Yes	Yes	Yes
Household fixed effects	Yes	Yes	Yes	Yes
Year fixed effects	Yes	Yes	Yes	Yes
Observations	49,037	25,812	16,535	3,946
R-squared	0.5250	0.4864	0.4869	0.5216

Table 10 shows that education debt positively affects household consumption upgrading in all four groups. The effect increases with higher financial literacy levels. Specifically, the very high financial literacy group exhibits the strongest effect. This suggests that higher financial literacy enhances the positive impact of education debt on consumption upgrading. Households with higher financial literacy possess better knowledge to weigh risks and benefits, leading to more rational and forward-looking decisions. As a result, they are more likely to direct education debt toward consumption upgrading through effective financial planning and decision-making.

#### 6.2.3 Heterogeneity in the classification of urban and rural households.

Given the dual economic structure of urban and rural areas, significant differences exist between them in terms of economic levels and education popularization [80]. Additionally, the urban-rural consumption divide is a key factor in China’s overall consumption gap and an important issue of consumption justice. Therefore, this paper examines the heterogeneous effects of education debt on urban and rural households separately. As shown in Table 11, columns (1) and (2), education debt positively impacts consumption upgrading for both urban and rural households, with both effects significant at the 1% level. This indicates that education debt significantly promotes development and enjoyment consumption in both groups.

**Table 11:** Heterogeneity analysis based on urban -rural household classification.

Variable	Consumption upgrading	
	Urban	Rural
	(1)	(2)
Total education debt(ln)	0.0046***	0.0058***
	(6.2674)	(7.3954)
Head of household control variables	Yes	Yes
Household control variables	Yes	Yes
Household fixed effects	Yes	Yes
Year fixed effects	Yes	Yes
Observations	66,352	33,473
R-squared	0.5491	0.4684

However, the impact of education debt on consumption upgrading is more pronounced in rural households. This may be because rural households typically have lower average incomes than urban households, making the potential income gains from education debt more impactful for them. This effect may help rural households move into the middle-income bracket, thereby driving a more significant upgrade in consumption. In contrast, urban households, with higher consumption levels and more diversified spending patterns, may experience a relatively smaller boost in consumption upgrading from education debt compared to rural households.

#### 6.2.4 Heterogeneity in household debt levels.

In this study, we measure household debt using the debt-to-income ratio, calculated by dividing total debt by disposable income. This ratio directly reflects a household’s debt servicing capacity. Table 12 shows the impact of education debt on consumption upgrading across households with different debt levels. We categorize households into three groups based on their debt-to-income ratios: the top 20% as high-leveraged households, the bottom 20% as low-leveraged households, and the remaining households as middle-leveraged households [81].

**Table 12:** Heterogeneity analysis based on household debt level.

Variable	Consumption upgrading		
	Low-leveraged	Middle-leveraged	High-leveraged
	(1)	(2)	(3)
Total education debt (ln)	0.0038*	0.0034***	0.0018
	(1.7774)	(2.9465)	(0.5496)
Head of household control variables	Yes	Yes	Yes
Household control variables	Yes	Yes	Yes
Household fixed effects	Yes	Yes	Yes
Year fixed effects	Yes	Yes	Yes
Observations	2,045	9,818	1,324
R-squared	0.5828	0.5643	0.6458

The results in column (2) of Table 12 indicate that education debt positively and significantly impacts consumption upgrading among middle-leveraged households. In contrast, column (3) shows no significant effect for high-leveraged households, likely because their small debt burden and high debt servicing capacity make education debt less impactful on consumption. Similarly, column (3) shows no significant effect for high-leveraged households, who may have adapted to high debt levels and possess more financial tools to manage their debts.

Overall, these findings suggest that middle-leveraged households, with their moderate risk tolerance, are more likely to use education debt to drive consumption upgrading.

## 7. Main conclusions and policy recommendations

### 7.1 Conclusion

Using five-period panel data from the China Household Finance Survey (CHFS) between 2013 and 2021, this study examines the impact of education debt on household consumption upgrading through a fixed-effects model. Results indicate that education debt significantly and positively affects total household consumption, particularly development and enjoyment consumption, thereby driving consumption upgrading. Robustness tests confirm the reliability of these findings. Mechanism analysis reveals that education debt promotes consumption upgrading primarily by boosting Internet consumption. However, this effect is weaker among risk-preference households. The heterogeneity analysis reveals that education debt exerts a more substantial positive effect on consumption among urban and rural households, households with high financial literacy, middle-and low-income groups, and middle-leveraged households.

This study demonstrates that education debt can significantly promote household consumption and drive consumption upgrading. Given China’s shift toward high-quality economic development, understanding these dynamics is crucial for formulating effective consumption policies and fostering a harmonious consumer market.

### 7.2 Policy implications

(1) **Enhancing education subsidies** The government should expand education subsidies and preferential loan policies, especially for low- and middle-income households. Establishing a special education fund to offer subsidized or low-interest education loans can alleviate the burden of education debt. Additionally, diversifying education financing channels, such as education bonds and trusts, can provide more options for households. Addressing urban-rural disparities through increased investment in rural education and simplifying loan and subsidy processes can also support consumption upgrading.
(2) **Strengthening household financial education** The government should expand education subsidies and preferential loan policies, particularly for low- and middle-income households. Establishing a special education fund to provide subsidized or low-interest loans could ease the burden of education debt. Additionally, diversifying education financing through instruments like education bonds and trusts would offer households more options. Addressing urban-rural disparities by increasing investment in rural education and simplifying loan and subsidy processes can further support consumption upgrading.
(3) **Implementing early warning systems for education debt.** Implementing early warning systems to monitor household debt can prevent over-indebtedness. Offering financial education courses and platforms with information on education costs, scholarships, and financial aid can support informed decision-making. Strengthening consumer protection and exploring household education investment insurance can safeguard households against risks, thereby enhancing consumption expectations and encouraging a shift from basic to development and enjoyment consumption.

The policy recommendations proposed in this study aim to establish a systematic institutional framework that effectively manages the potential risks of education debt while fully leveraging its dual role in promoting consumption upgrading and human capital accumulation, thereby providing a new policy anchor for China’s high-quality economic development.

## Supporting information

S1 Appendix Statistical overview of education debt. (PDF)

S2 Appendix Data cleaning statistics. (PDF)

S3 Appendix Balance test. (PDF)

S1 Fig The flow chart of this research. (PDF)

S2 Fig Benchmark regression visualization. (PDF)

S3 Fig Common support condition. (PDF)

## References

[1] Wang Q, Luo X, Yang J. Understanding China’s double reduction policy on educational economy. Glob Econ Observ. 2022;10(1):63–9.

[2] Yi S, Qi Y, Ya Y, Shi J, Cui Y. The impact of China’s digital inclusive financial development gap on the optimization of rural consumption structure. PLoS One. 2024;19(8):e0308412. doi: 10.1371/journal.pone.0308412 39116100 PMC11309468

[3] Hanushek EA, Woessmann L. The role of cognitive skills in economic development. J Econ Lit. 2008;46(3):607–68. doi: 10.1257/jel.46.3.607

[4] Dynarski SM. Does aid matter? Measuring the effect of student aid on college attendance and completion. Am Econ Rev. 2003;93(1):279–88. doi: 10.1257/000282803321455287

[5] Callender C, Mason G. Does student loan debt deter higher education participation? New evidence from England. Ann Am Acad Pol Soc Sci. 2017;671(1):20–48. doi: 10.1177/0002716217696041

[6] Debande O. A review of instruments for student loans in tertiary education. Euro J Educ. 2004;39(2):161–90. doi: 10.1111/j.1465-3435.2004.00174.x

[7] Abdelzaher MA. A comparative study between informal and formal finance: a literature review. AFR. 2019;8(4):231. doi: 10.5430/afr.v8n4p231

[8] Turvey CG, Kong R. Informal lending amongst friends and relatives: Can microcredit compete in rural China?. China Econ Rev. 2010;21(4):544–56. doi: 10.1016/j.chieco.2010.05.001

[9] Chi W, Qian X. Human capital investment in children: An empirical study of household child education expenditure in China, 2007 and 2011. China Econ Rev. 2016;37:52–65. doi: 10.1016/j.chieco.2015.11.008

[10] Yang R. Transnational higher education in China: contexts, characteristics and concerns. Aust J Educ. 2008;52(3):272–86. doi: 10.1177/000494410805200305

[11] Chen Y, Yuan M, Zhang M. Income inequality and educational expenditures on children: evidence from the China Family Panel Studies. China Econ Rev. 2023;78:101932. doi: 10.1016/j.chieco.2023.101932

[12] Fan L, Chatterjee S. Financial socialization, financial education, and student loan debt. J Fam Econ Iss. 2018;40(1):74–85. doi: 10.1007/s10834-018-9589-0

[13] Bowie-Viverette AC, Saulnier S. The student loan debt crisis: a narrative review. J Hum Rights Soc Work. 2023;9(1):3–9. doi: 10.1007/s41134-023-00281-0

[14] Zhang X, He Y. Influence of educational attainment on consumption. Front Educ China. 2007;2(2):259–72. doi: 10.1007/s11516-007-0022-y

[15] Chen F, Mao S, Huang R. Age structure of the population and household consumption expenditure on tourism. Finance Res Lett. 2024;60:104896. doi: 10.1016/j.frl.2023.104896

[16] Piao Y, Li M, Sun H, Yang Y. Income inequality, household debt, and consumption growth in the United States. Sustainability. 2023;15(5):3910. doi: 10.3390/su15053910

[17] Lettau M, Ludvigson SC. Understanding trend and cycle in asset values: reevaluating the wealth effect on consumption. Am Econ Rev. 2004;94(1):276–99. doi: 10.1257/000282804322970805

[18] Berisha E, Meszaros J. Household debt, consumption, and income inequality. Int Econ J. 2018;32(2):161–76. doi: 10.1080/10168737.2018.1481874

[19] Barro RJ, Lee JW. A new data set of educational attainment in the world, 1950–2010. J Dev Econ. 2013;104:184–98. doi: 10.1016/j.jdeveco.2012.10.001

[20] Campbell JY, Cocco JF. How do house prices affect consumption? Evidence from micro data. J Monet Econ. 2007;54(3):591–621. doi: 10.1016/j.jmoneco.2005.10.016

[21] Han H, Si F. How does the composition of asset portfolios affect household consumption: evidence from China based on micro data. Sustainability. 2020;12(7):2946. doi: 10.3390/su12072946

[22] Wang Y, Zhao W, Meng W. Bilateral effect of aging population on consumption structure: evidence from China. Front Public Health. 2022;10:941485. doi: 10.3389/fpubh.2022.941485 36091508 PMC9452717

[23] Guo L, Wang F. The impact of demographic dividend shift on household consumption:evidence from China. Humanit Soc Sci Commun. 2024;11(1). doi: 10.1057/s41599-024-04251-3

[24] Carroll CD, Slacalek J, Sommer M. International evidence on sticky consumption growth. Rev Econ Stat. 2011;93(4):1135–45. doi: 10.1162/rest_a_00122

[25] Beliaeva T, Ferasso M, Kraus S, Mahto RV. Marketing and family firms: theoretical roots, research trajectories, and themes. J Business Res. 2022;144:66–79. doi: 10.1016/j.jbusres.2022.01.094

[26] Shi MM, Jiang Z, Zhou XY. Consumption upgrading or downgrading. China Ind Econ. 2019;(07):42–60. doi: 10.19581/j.cnki.ciejournal.2019.07.003

[27] Liu L, Wang Q, Zhang A. The impact of housing price on non-housing consumption of the Chinese households: a general equilibrium analysis. North Am J Econ Financ. 2019;49:152–64. doi: 10.1016/j.najef.2019.04.010

[28] Shen ZL. Household debts and the quality of consumption: evidence from the regulatory effect of income inequality. J Macro-Qual Res. 2021;9(03):114–28. doi: 10.13948/j.cnki.hgzlyj.2021.03.009

[29] Min W, Yu J, Wu J. The role of education in expanding domestic demand to drive economic growth. Educational research in China. Springer Nature Singapore; 2024. p. 435–52. doi: 10.1007/978-981-97-0277-0_18

[30] Modigliani F, Brumberg R. Utility analysis and the consumption function: an interpretation of cross-section data 1. In Post-keynesian economics. Routledge; 2013. p. 388–436. doi: 10.7551/mitpress/1923.003.0004

[31] Friedman M. Theory of the consumption function. Princeton University Press; 2018. doi: 10.2307/j.ctv39x7zh

[32] Carroll CD, Kimball MS. On the concavity of the consumption function. Econometrica. 1996;64(4):981. doi: 10.2307/2171853

[33] Hall RE. Stochastic implications of the life cycle-permanent income hypothesis: theory and evidence. J Pol Economy. 1978;86(6):971–87. doi: 10.1086/260724

[34] Hou XX, Fu JL. Does income uncertainty influence family education expenditure? Rev Econ Manag. 2023;39(03):88–101. doi: 10.13962/j.cnki.37-1486/f.2023.03.006

[35] Li H, Huang L. Health, education, and economic growth in China: empirical findings and implications. China Econ Rev. 2009;20(3):374–87. doi: 10.1016/j.chieco.2008.05.001

[36] Beshears J, Choi JJ, Laibson D, Madrian BC. Behavioral household finance. Handbook of behavioral economics: applications and foundations 1. Elsevier; 2018. p. 177–276. doi: 10.1016/bs.hesbe.2018.07.004

[37] Seginer R, Vermulst A. Family environment, educational aspirations, and academic achievement in two cultural settings. J Cross-Cult Psychol. 2002;33(6):540–58. doi: 10.1177/00220022102238268

[38] Chen Y, Yang W, Hu Y. Internet development, consumption upgrading and carbon emissions-an empirical study from China. Int J Environ Res Public Health. 2022;20(1):265. doi: 10.3390/ijerph20010265 36612587 PMC9819726

[39] Zhao L, Fu B, Bai S. Understanding the influence of personalized recommendation on purchase intentions from a self-determination perspective: contingent upon product categories. JTAER. 2025;20(1):32. doi: 10.3390/jtaer20010032

[40] Acevedo A. A personalistic appraisal of Maslow’s needs theory of motivation: from “Humanistic” psychology to integral humanism. J Bus Ethics. 2015;148(4):741–63. doi: 10.1007/s10551-015-2970-0

[41] Hong M, Wang J, Tian M. Rural social security, precautionary savings, and the upgrading of rural residents’ consumption structure in China. Sustainability. 2022;14(19):12455. doi: 10.3390/su141912455

[42] Kwak EJ, Grable JE. A comparison of financial risk-tolerance assessment methods in predicting subsequent risk tolerance and future portfolio choices. Risks. 2024;12(11):170. doi: 10.3390/risks12110170

[43] Jin C, Mao W, Jin K, Yang Q, Zang J, Chen R. Factors influencing household debt structure under various credit constraints. J Manag Sci Eng. 2025;10(1):97–110. doi: 10.1016/j.jmse.2024.08.003

[44] Li XN, Li R. Consumption structure and influence factors of rural residents in four economic regions of China. J Quan Technol Econ. 2013;30(09):89–105. doi: 10.13653/j.cnki.jqte.2013.09.018

[45] Bao W, Zhao CM. Theoretical analysis and empirical evidence demonstrate the role of employment quality in promoting household consumption upgrading. Theory Pract Financ Econ. 2024;45(02):121–7. doi: 10.16339/j.cnki.hdxbcjb.2024.02.015

[46] Lu Q, Hua J. Micro-household human capital investment decisions and a simulation study from the intergenerational conflict perspective. Int J Environ Res Public Health. 2023;20(3):1696. doi: 10.3390/ijerph20031696 36767062 PMC9913944

[47] López-Madrigal C, de la Fuente J, García-Manglano J, Martínez-Vicente JM, Peralta-Sánchez FJ, Amate-Romera J. The role of gender and age in the emotional well-being outcomes of young adults. Int J Environ Res Public Health. 2021;18(2):522. doi: 10.3390/ijerph18020522 33435219 PMC7828022

[48] Ou S-R, Yoo S, Reynolds AJ. Educational growth trajectories in adulthood: Findings from an inner-city cohort. Dev Psychol. 2021;57(7):1163–78. doi: 10.1037/dev0001198 34435830 PMC8406409

[49] Ndayambaje E, Pierewan AC, Nizeyumukiza E, Nkundimana B, Ayriza Y. Marital status and subjective well-being: does education level take into account? CP. 2020;39(1):120–32. doi: 10.21831/cp.v39i1.29620

[50] DeSalvo KB, Bloser N, Reynolds K, He J, Muntner P. Mortality prediction with a single general self-rated health question. A meta-analysis. J Gen Intern Med. 2006;21(3):267–75. doi: 10.1111/j.1525-1497.2005.00291.x 16336622 PMC1828094

[51] Giugni M, Grasso M. Party membership and social movement activism: a macro–micro analysis. Party Politics. 2019;27(1):92–102. doi: 10.1177/1354068818823446

[52] Blanden J. Family income and educational attainment: a review of approaches and evidence for Britain. Oxford Rev Econ Policy. 2004;20(2):245–63. doi: 10.1093/oxrep/grh014

[53] Lockwood J, Webber D. Non-completion, student debt, and financial well-being: evidence from the survey of household economics and decisionmaking. FEDS Notes. 2023;(8/21/2023):None-None. doi: 10.17016/2380-7172.3371

[54] Adongo AA, Dapaah JM, Wireko D. The influence of family size on academic performance of high school students in Ghana. SN Soc Sci. 2022;2(9). doi: 10.1007/s43545-022-00478-6

[55] Bogan VL. Household asset allocation, offspring education, and the sandwich generation. Am Econ Rev. 2015;105(5):611–5. doi: 10.1257/aer.p20151115

[56] An empirical study on the effect of child dependency ratio on household consumption—Focusing on the “two-child policy” from 2016. FSST. 2022;4(6). doi: 10.25236/fsst.2022.040611

[57] Emerson P, Knabb S, Sirbu A-I. Does the old-age dependency ratio place a drag on secular growth?. Econ Analy Policy. 2024;82:1056–70. doi: 10.1016/j.eap.2024.04.026

[58] Zhang Q, Yuan Y. A study on the impact of household head’s employment modes on the living standards of rural migrant workers’ families. PLoS One. 2024;19(12):e0312518. doi: 10.1371/journal.pone.0312518 39621740 PMC11611100

[59] Zhang ZR, Zhu W. Has the household debt inhibited the consumption upgrading in China? ——Evidence from the China family panel studies. J Modern Finance. 2019;24(08):34–44. doi: 10.16529/j.cnki.11-4613/f.2019.08.005

[60] Xu M, Jiang Y. Can the China’s industrial structure upgrading narrow the gap between urban and rural consumption? J Quant Technol Econ. 2015;32(03):3–21. doi: 10.13653/j.cnki.jqte.2015.03.001

[61] Zhan Z, Su Z-W, Chang H-L. Education and quality of life: does the internet matter in China?. Front Public Health. 2022;10:860297. doi: 10.3389/fpubh.2022.860297 35372198 PMC8971524

[62] Haleem A, Javaid M, Qadri MA, Suman R. Understanding the role of digital technologies in education: a review. Sustain Oper Comput. 2022;3:275–85. doi: 10.1016/j.susoc.2022.05.004

[63] Wang Y, Peng C, Cai S. Does parents’ cognitive ability affect household educational investment? Evidence from Chinese families with left behind children. PLoS One. 2023;18(6):e0286987. doi: 10.1371/journal.pone.0286987 37384646 PMC10310008

[64] Berry JE. The internet: an educational system for equalizing educational opportunity. Handbook on promoting social justice in education. Springer International Publishing; 2020. p. 1587–607. doi: 10.1007/978-3-030-14625-2_74

[65] Hu N, Hou G. Mobile payment, digital inclusive finance, and residents’ consumption behavior research. PLoS One. 2024;19(7):e0288679. doi: 10.1371/journal.pone.0288679 39037958 PMC11262667

[66] Xiaoying Z, Kaili K. Internet development, spatial spillover and upgrading of china’s consumption structure: a spatial econometrics analysis based on data of 242 cities during 2005-2016. E3S Web Conf. 2021;251:01037. doi: 10.1051/e3sconf/202125101037

[67] Tian Z, Wang R, Tan Y. Research on the influence mechanism of internet use on rural residents’ consumption level in China--The mediating effect of consumption literacy. PLoS One. 2023;18(11):e0294723. doi: 10.1371/journal.pone.0294723 38011127 PMC10681202

[68] Zeng JH, Zhong RY. Does Internet Contribute to the consumption upgrading of residents? —Research based on city consumption search index in guangdong province. Economist. 2021;(08):31–41.

[69] Campbell JY, Viceira LM. Strategic asset allocation. Oxford University PressOxford; 2002. doi: 10.1093/0198296940.001.0001

[70] Hu D, Zhai C, Wang X, Huang X. Does household risk attitude affect consumption expenditure? Evidence from Chinese urban households. Econ Model. 2024;141:106924. doi: 10.1016/j.econmod.2024.106924

[71] Setterfield M, Kim YK. How financially fragile can households become? Household borrowing, the welfare state, and macroeconomic resilience. Rev Evol Polit Econ. 2023;5(1):121–51. doi: 10.1007/s43253-023-00106-w

[72] Banerjee AV, Duflo E. The economic lives of the poor. J Econ Perspect. 2007;21(1):141–67. doi: 10.1257/jep.21.1.141 19212450 PMC2638067

[73] Deng Y, Kang J, Wang Y, Hu Z, Gong W. Effects of debt dynamics and financial literacy on consumer market behavior. Financ Res Lett. 2025;72:106541. doi: 10.1016/j.frl.2024.106541

[74] Hardy BL, Marcotte DE. Education and the dynamics of middle-class status. 2020. https://api.semanticscholar.org/CorpusID:225820487

[75] Galvez R, Ignacio R. Scholarship grantees ‘satisfaction on student financial aid unit and its effects on scholars’ financial stress. Cosmos: An International Journal of Art and Higher Education. 2023;12(2):16–22. doi: 10.46360/cosmos.ahe.520232003

[76] Killewald A, Pfeffer FT, Schachner JN. Wealth inequality and accumulation. Annu Rev Sociol. 2017;43:379–404. doi: 10.1146/annurev-soc-060116-053331 28798522 PMC5546759

[77] Ni J, Gao Y. Opportunities for higher education for children, financial literacy and household capital. Financ Res Lett. 2025;78:107236. doi: 10.1016/j.frl.2025.107236

[78] Lusardi A, Mitchell OS. The economic importance of financial literacy: theory and evidence. J Econ Lit. 2014;52(1):5–44. doi: 10.1257/jel.52.1.5 28579637 PMC5450829

[79] Agarwal S, Driscoll JC, Gabaix X, Laibson D. The age of reason: financial decisions over the life cycle and implications for regulation. eca. 2009;2009(2):51–117. doi: 10.1353/eca.0.0067

[80] Chen X, Wu R. How can rural industrial revitalization and rural education level reduce the urban–rural income gap? Financ Res Lett. 2025;73:106592. doi: 10.1016/j.frl.2024.106592

[81] Sala H, Trivin P. Household finances, debt overhang and consumption patterns. Econ Model. 2024;139:106836. doi: 10.1016/j.econmod.2024.106836

