# Supplementary material for: Education debt and household consumption upgrading: Positive incentives or inhibitions?
Source: PLoS One. 2025 Oct 13;20(10):e0332318. doi: 10.1371/journal.pone.0332318 (PMC12517517; doi:10.1371/journal.pone.0332318)
Supplement: S1 Appendix — (PDF) [file pone.0332318.s001.pdf]

S1 Appendix: Statistical overview of education debt

Table 1 Statistical overview of education debt

Variable	N	Mean	Median	Std.Dev	Min	Max
Education debt dummy	100608	0.0302	0	0.1712	0	1
Total education debt	100608	391.15	0	2536.93	0	20000
Total education debt(ln)	100608	0.2770	0	1.5794	0	9.9035

① The mean of the total amount of education debt before logarithmic transformation（391.15）、Median（0）、Std. Dev（2536.93）、Min（0）and Max（20000）
② The mean of the total amount of education debt after logarithmic transformation（0.2770）、Median（0）、Std. Dev（1.5794）、Min（0）and Max（9.9035）

Logarithmic transformation verification:
The original data shows a severe right bias: the mean total education debt (391.15) is greater than the median (0), the standard deviation (2536.93) is 6.5 times the mean, and the maximum value (20000) is 52 times the mean. The ratio of the standard deviation (1.5794) to the mean (0.2770) after logarithmic transformation decreased from 6.5 to 5.5, indicating that the degree of dispersion of the variable has been improved.

Statistically speaking：
The total amount of education debt (the independent variable) and consumption upgrading (the dependent variable) are both treated logarithmically. The regression coefficient β of the total education debt can be directly interpreted as an elasticity coefficient, with its economic meaning being that when education debt changes by 1%, consumption upgrading changes by β %.
