# Supplementary material for: Education debt and household consumption upgrading: Positive incentives or inhibitions?
Source: PLoS One. 2025 Oct 13;20(10):e0332318. doi: 10.1371/journal.pone.0332318 (PMC12517517; doi:10.1371/journal.pone.0332318)
Supplement: S2 Appendix — (PDF) [file pone.0332318.s002.pdf]

S2 Appendix: Data Cleaning Statistics

Table 1 Descriptive statistics of the main variables before data cleaning

Variable	N	Mean	Median	Std. Dev	Min	Max
**Dependent variables**						
Total household consumption	158750	71125.3	47424	550833.8	0	1.70e+08
Total household consumption(ln)	158750	10.7180	10.7669	0.9348	0	18.9486
Subsistence consumption	158750	34275.3	25350	157298.9	0	6.00e+07
Subsistence consumption(ln)	158750	10.0252	10.1406	0.9966	0	17.9098
Development and enjoyment consumption	158750	36849.9	18700	525833.7	0	1.70e+08
Development and enjoyment consumption(ln)	158750	9.7553	9.8363	1.2634	0	18.9485
Consumption upgrading	158719	0.4445	0.4376	0.2073	0	1
**Core independent variables**						
Education debt dummy	158750	0.0297	0	0.1697	0	1
Total education debt	158750	817.2751	0	16053.1	0	5000000
Total education debt(ln)	158750	0.2810	0	1.6217	0	15.4249
**Control variables**						
Gender dummy	158750	0.7534	1	0.4311	0	1
Age	158692	53.6011	53	14.1370	0	116
$Age^2/100$	158750	30.7294	28.09	15.3302	0	134.56
Year of education	158750	9.2519	9	4.1506	0	22
Marital dummy	158750	0.8506	1	0.3564	0	1
Health dummy	158750	0.7559	1	0.4295	0	1
Party member dummy	158750	0.4001	0	0.4899	0	1
Household income（ln）	155232	10.2931	10.7790	2.1367	-0.0001	16.3763
Household size	158750	3.3430	3	1.7383	1	27
Household assets（ln）	158748	12.5929	12.7986	1.8359	0	30.6268
Housing debts（ln）	158750	1.6228	0	4.0207	0	17.0341
Child dependency ratio	158750	0.1299	0	0.1731	0	1
Elderly dependency ratio	158750	0.3048	0	0.3926	0	2
Household employment rate	158750	0.4727	0.5	0.3370	0	1.3333

Table 2 Descriptive statistics of the main variables after data cleaning (eliminating missing values)

Variable	N	Mean	Median	Std. Dev	Min	Max
**Dependent variables**						
Total household consumption	155139	71238.5	47600	557017.7	3	1.70e+08
Total household consumption(ln)	155139	10.7228	10.7706	0.9209	1.3863	18.9486
Subsistence consumption	155139	34295.9	25420	158937.9	0	6.00e+07
Subsistence consumption(ln)	155139	10.0300	10.1433	0.9842	0	17.9098
Development and enjoyment consumption	155139	36943.5	18770	531831.7	0	1.70e+08
Development and enjoyment consumption(ln)	155139	9.7598	9.8401	1.2551	0	18.9485
Consumption upgrading	155139	0.4444	0.4375	0.2073	0	1
**Core independent variables**						
Education debt dummy	155139	0.0291	0	0.1682	0	1
Total education debt	155139	808.257	0	16185	0	5000000
Total education debt(ln)	155139	0.2759	0	1.6078	0	15.4249
**Control variables**						
Gender dummy	155139	0.7518	1	0.4320	0	1
Age	155139	53.6547	54	14.1838	0	116
$Age^2/100$	155139	30.8001	29.16	15.3844	0	134.56
Year of education	155139	9.2807	9	4.1541	0	22
Marital dummy	155139	0.8496	1	0.3574	0	1
Health dummy	155139	0.7573	1	0.4287	0	1
Party member dummy	155139	0.4015	0	0.4902	0	1
Household income（ln）	155139	10.2944	10.7789	2.1347	0	16.3763
Household size	155139	3.3365	3	1.7355	1	27
Household assets（ln）	155139	12.6018	12.8096	1.8351	0	30.6268
Housing debts（ln）	155139	1.6252	0	4.0254	0	17.0344
Child dependency ratio	155139	0.1288	0	0.1719	0	1
Elderly dependency ratio	155139	0.3070	0	0.3936	0	2
Household employment rate	155139	0.4702	0.5	0.3372	0	1.3333

Table 3 Descriptive statistics of the main variables after data cleaning (excluding outliers)

Variable	N	Mean	Median	Std. Dev	Min	Max
**Dependent variables**						
Total household consumption	100608	69448.2	47983.5	389221.7	3	1.20e+08
Total household consumption(ln)	100608	10.7326	10.7786	0.8974	1.3863	18.6036
Subsistence consumption	100608	33343.1	25400	41877.8	0	1912000
Subsistence consumption(ln)	100608	10.0339	10.1425	0.9379	0	14.4637
Development and enjoyment consumption	100608	36105.1	19350	384364	0	1.20e+08
Development and enjoyment consumption(ln)	100608	9.7954	9.8705	1.2049	0	18.6034
Consumption upgrading	100608	0.4508	0.4452	0.2040	0	1
**Core independent variables**						
Education debt dummy	100608	0.0302	0	0.1711	0	1
Total education debt	100608	764.8323	0	9324.5	0	1200000
Total education debt(ln)	100608	0.2847	0	1.6292	0	13.9978
**Control variables**						
Gender dummy	100608	0.7683	1	0.4219	0	1
Age	100608	53.5155	53	13.5585	18	112
$Age^2/100$	100608	30.4774	28.09	14.7519	3.24	125.44
Year of education	100608	9.4314	9	3.9847	0	22
Marital dummy	100608	0.8696	1	0.3367	0	1
Health dummy	100608	0.7603	1	0.4269	0	1
Party member dummy	100608	0.3628	0	0.4808	0	1
Household income（ln）	100608	10.3674	10.8158	2.0541	0	16.3106
Household size	100608	3.4504	3	1.7513	1	21
Household assets（ln）	100608	12.6956	12.8744	1.7517	0	20.4139
Housing debts（ln）	100608	1.6277	0	4.0225	0	17.0344
Child dependency ratio	100608	0.1320	0	0.1706	0	1
Elderly dependency ratio	100608	0.2924	0	0.3824	0	1
Household employment rate	100608	0.4716	0.5	0.3296	0	1

Table 4 Descriptive statistics of the main variables after winsorization

Variable	N	Mean	Median	Std. Dev	Min	Max
**Dependent variables**						
Total household consumption	100608	65655.6	47983.5	62440.0	4618	373759.9
Total household consumption(ln)	100608	10.7333	10.7786	0.8688	8.4379	12.8314
Subsistence consumption	100608	32043.8	25400	28504.6	2050	179300
Subsistence consumption(ln)	100608	10.0426	10.1425	0.8528	7.6261	12.0968
Development and enjoyment consumption	100608	32889.5	19350	41579.9	760	256000
Development and enjoyment consumption(ln)	100608	9.8004	9.8705	1.1541	6.6346	12.4529
Consumption upgrading	100608	0.4508	0.4452	0.2039	0	1
**Core independent variables**						
Education debt dummy	100608	0.0302	0	0.1712	0	1
Total education debt	100608	391.15	0	2536.9	0	20000
Total education debt(ln)	100608	0.2770	0	1.5794	0	9.9035
**Control variables**						
Gender dummy	100608	0.7683	1	0.4219	0	1
Age	100608	53.5155	53	13.5585	18	112
$Age^2/100$	100608	30.4774	28.09	14.7519	3.24	125.44
Year of education	100608	9.4315	9	3.9847	0	22
Marital dummy	100608	0.8696	1	0.3367	0	1
Health dummy	100608	0.7603	1	0.4269	0	1
Party member dummy	100608	0.3628	0	0.4808	1	1
Household income（ln）	100608	10.3611	10.8158	2.0434	0	13.3068
Household size	100608	3.4504	3	1.7513	7.6829	21
Household assets（ln）	100608	12.7044	12.8744	1.6952	0	16.6070
Housing debts（ln）	100608	1.6222	0	4.0057	0	13.3573
Child dependency ratio	100608	0.1309	0	0.1676	0	0.5
Elderly dependency ratio	100608	0.2924	0	0.3824	0	1
Household employment rate	100608	0.4716	0.5	0.3296	0	1
