# Supplementary material for: Education debt and household consumption upgrading: Positive incentives or inhibitions?
Source: PLoS One. 2025 Oct 13;20(10):e0332318. doi: 10.1371/journal.pone.0332318 (PMC12517517; doi:10.1371/journal.pone.0332318)
Supplement: S3 Appendix — (PDF) [file pone.0332318.s003.pdf]

S3 Appendix: Balance test

Control variables	Sample category	Mean		%Bias	T-test	
		Treated	Control		t	P>|t|
Gender dummy	U	0.802	0.766	8.8	7.61	0.001
	M	0.802	0.796	1.4	0.95	0.341
Age	U	47.589	54.023	-48.3	-42.79	0.000
	M	47.589	47.534	0.4	0.27	0.788
$Age^2/100$	U	24.394	30.984	-47.2	-40.30	0.000
	M	24.394	24.469	-0.5	-0.37	0.713
Year of education	U	10.831	9.316	37.2	34.17	0.000
	M	10.831	10.89	-1.5	-0.95	0.343
Marital dummy	U	0.859	0.871	-3.4	-3.08	0.002
	M	0.859	0.854	1.7	1.07	0.286
Health dummy	U	0.806	0.756	12.3	10.58	0.000
	M	0.806	0.803	0.9	0.61	0.540
Party member dummy	U	0.337	0.365	-5.7	-5.02	0.000
	M	0.337	0.342	-0.9	-0.59	0.553
Household income(ln)	U	10.736	10.333	19.8	17.67	0.000
	M	10.736	10.727	0.5	0.31	0.754
Household size	U	3.576	3.443	7.7	6.81	0.000
	M	3.576	3.532	2.5	1.71	0.088
Household assets(ln)	U	13.142	12.671	27.7	24.91	0.000
	M	13.142	13.123	1.1	0.72	0.471
Housing debts(ln)	U	2.377	1.553	19.9	18.35	0.000
	M	2.377	2.469	-2.1	-1.26	0.208
Child dependency ratio	U	0.162	0.128	19.9	18.08	0.000
	M	0.162	0.161	0.5	0.33	0.741
Elderly dependency ratio	U	0.173	0.302	-37.2	-30.24	0.000
	M	0.173	0.184	-3.2	-2.34	0.020
Household employment rate	U	0.519	0.468	16.2	13.87	0.000
	M	0.519	0.519	-0.2	-0.12	0.908

The table above presents the results of the balance test after propensity score matching (PSM) has been performed on the sample. To mitigate the endogeneity bias caused by data bias and variable issues within the sample, the propensity score matching method (PSM) was employed. Specifically, the propensity of households to be risk-averse was used as a quasi-natural experiment, with risk-averse household serving as the treatment group and risk-tolerant household as the control group. Nearest neighbor matching (1:4) was applied to match the sample, making it more balanced and further constructing a new sample to test the benchmark regression results. When conducting PSM analysis, a balance test should be performed, and the results are shown in the table above. The results indicate that the P-values of all variables after matching are generally

greater than 0.1, meaning that there is no significant difference between the treatment and control groups after propensity score matching. The standard deviations of all variables after matching are less than 5%, and almost all observations fall within the common support range. The PSM method results in minimal sample loss. The balance test results show that this method is reasonable. The regression results after matching are shown in the first column of Table 5 in the main text, indicating that education debt has a significant positive effect on household consumption upgrading, which is significant at the 1% level.
