# Supplementary figures and images for: Education debt and household consumption upgrading: Positive incentives or inhibitions?
Source: PLoS One. 2025 Oct 13;20(10):e0332318. doi: 10.1371/journal.pone.0332318 (PMC12517517; doi:10.1371/journal.pone.0332318)

S1 Figure

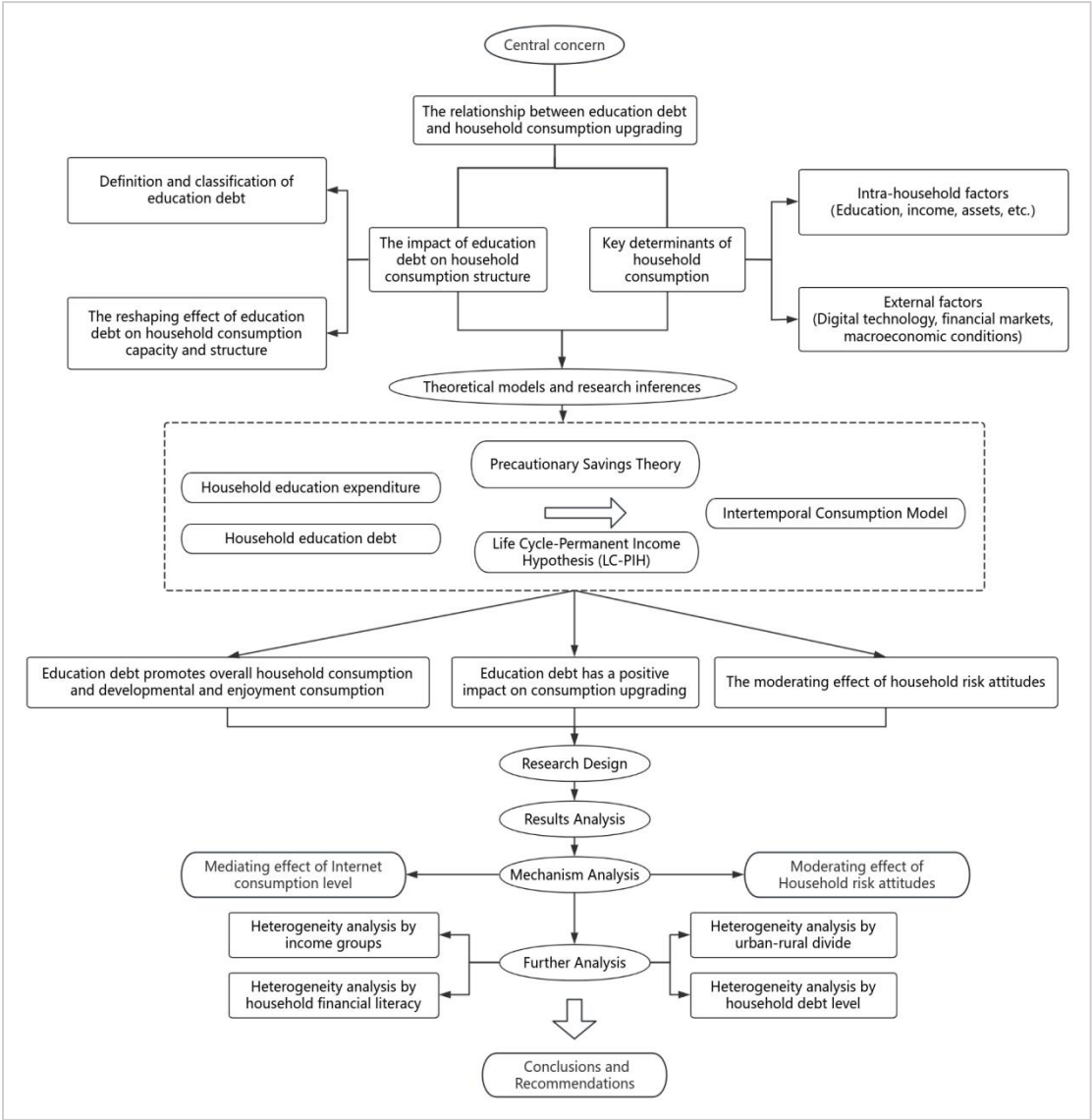

Supplement: S1 Fig — (PDF) [file pone.0332318.s004.pdf]
