# Supplementary material for: Education debt and household consumption upgrading: Positive incentives or inhibitions?
Source: PLoS One. 2025 Oct 13;20(10):e0332318. doi: 10.1371/journal.pone.0332318 (PMC12517517; doi:10.1371/journal.pone.0332318)
Supplement: S2 Fig — (PDF) [file pone.0332318.s005.pdf]

S2 Figure

A. Forest plot

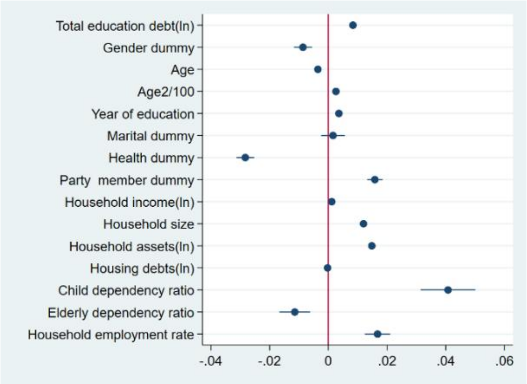

B. Scatter diagram and fitted line

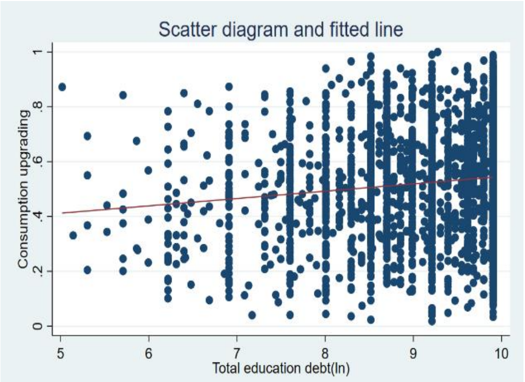

Figure A is a visualization of the basic regression of the impact of education debt on household consumption upgrading in this study. The length of each short horizontal line represents the range of the confidence interval. If it does not intersect with the middle horizontal axis at 0, it indicates that the regression coefficient of the relevant variable is significant. Therefore, it can be seen that education debt has a significant positive effect on household consumption upgrading.

Figure B is a scatter diagram and fitted line of the relationship between education debt and household consumption upgrading. Based on the fitted regression line drawn for each point, it is found that there is indeed a positive linear relationship between the two, indicating that education debt has a certain promoting effect on household consumption upgrading.
