# Supplementary material for: Education debt and household consumption upgrading: Positive incentives or inhibitions?
Source: PLoS One. 2025 Oct 13;20(10):e0332318. doi: 10.1371/journal.pone.0332318 (PMC12517517; doi:10.1371/journal.pone.0332318)
Supplement: S3 Fig — (PDF) [file pone.0332318.s006.pdf]

S3 Figure

A. Before sample matching B. After sample matching

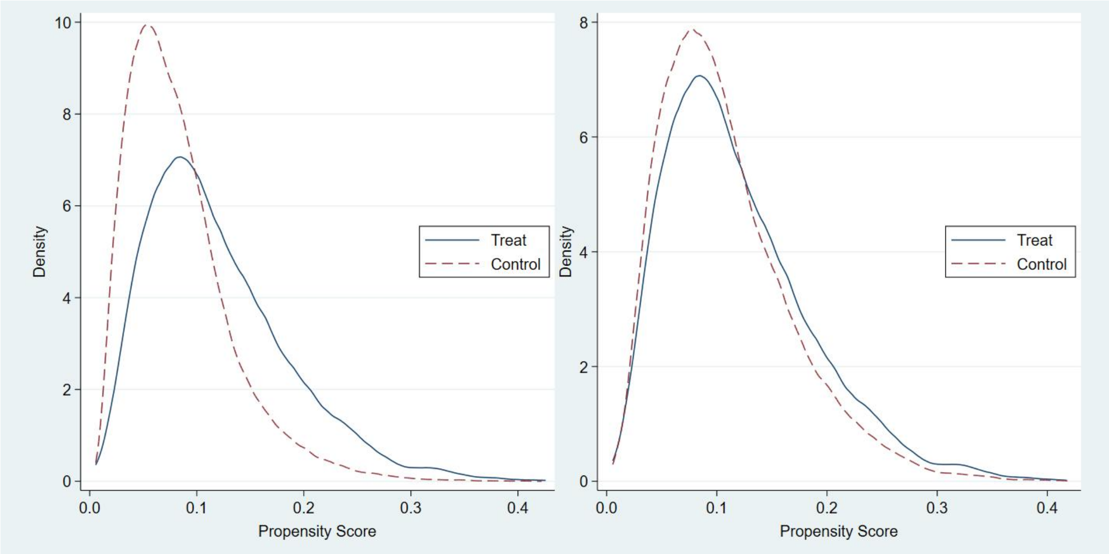

To compare the differences in propensity score values between the processing group and the control group before and after matching, we plotted the corresponding kernel density function graphs. Compared with Figure A, the density function graphs of the control group and the processing group in Figure B are closer, indicating that the features of the processing group and the control group are more similar after matching, thereby conducting a robustness test for the study in this paper.
